# Supplementary material for: Gene-level alignment of single-cell trajectories
Source: Nat Methods. 2024 Sep 19;22(1):68–81. doi: 10.1038/s41592-024-02378-4 (PMC11725504; doi:10.1038/s41592-024-02378-4)
Supplement: Supplementary file 1 — Supplementary Figs. 1–7, Table 1 and an outline of Tables 2–10 and Data, Note and Tables 2–10. [file 41592_2024_2378_MOESM1_ESM.pdf]

nature methods

Supplementary information https://doi.org/10.1038/s41592-024-02378-4

Gene-level alignment of single-cell trajectories

In the format provided by the authors and unedited

Supplementary Information

For manuscript:

Gene-level alignment of single cell trajectories

Contents

Supplementary Figures.......... 2
Supplementary Tables.......... 14
Supplementary Note.......... 17
References.......... 28

Supplementary Figures

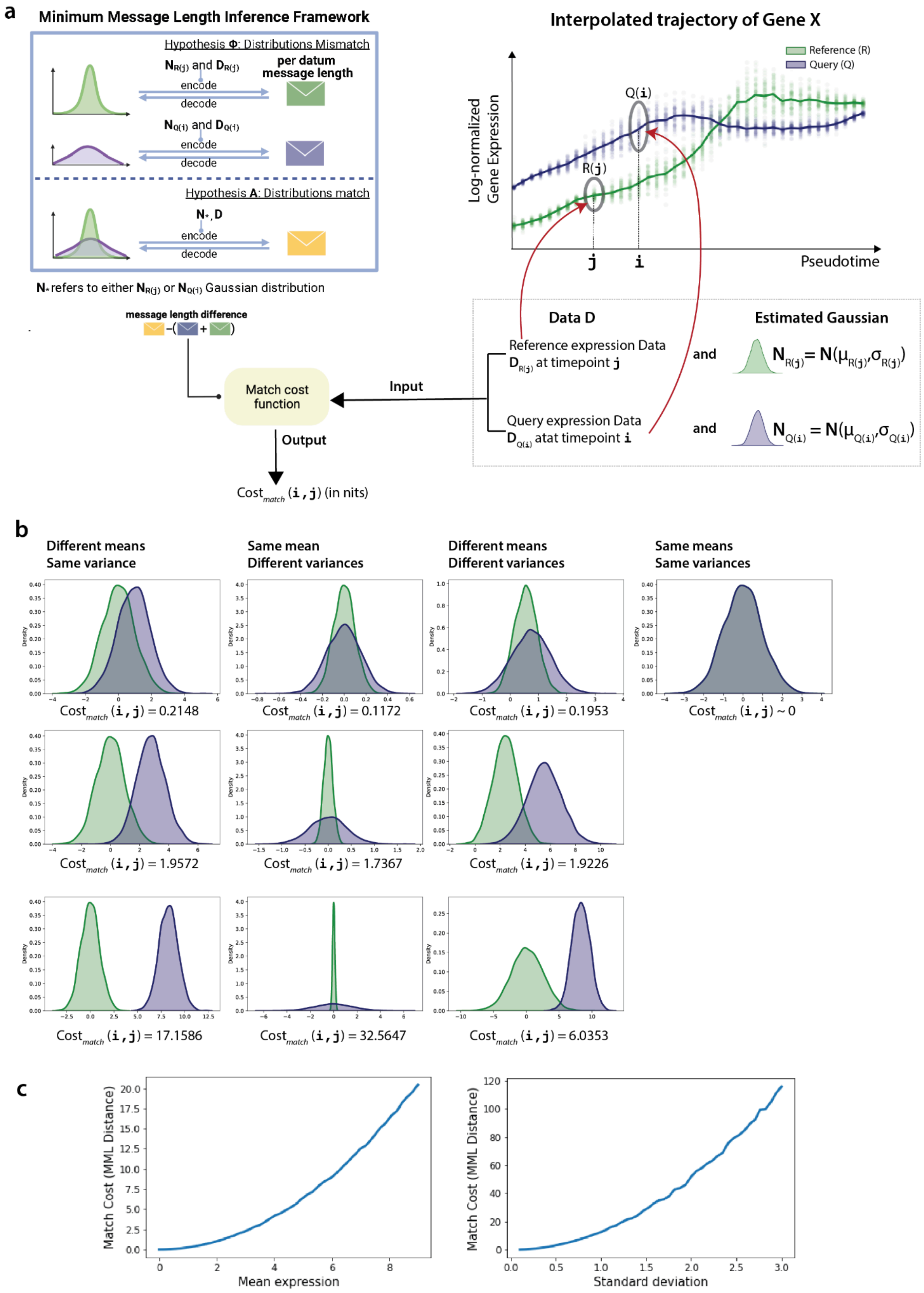

Supplementary Fig. 1 | Minimum message length (MML) inference based distance function to compute $Cost_{match}(i, j)$ of a reference (R) time point R_j and query (Q) time point Q_i. a, Top-right: interpolated log-normalized expression (y-axis) of gene X in the reference and query single-cells along pseudotime (x-axis). Bold lines represent mean expression trends; Faded data points represent interpolated data (50 random samples from estimated Gaussian distribution at each time point). G2G algorithm computes the cost of matching every R and Q time point pair using their interpolated expression distributions. Consider R_j and Q_i time points and their respective single-cell data for X, $R(j)$ and $Q(i)$, as circled in the plot. Their interpolated expression distributions are $N(\mu_{R(j)}, \sigma_{R(j)})$ and $N(\mu_{Q(i)}, \sigma_{Q(i)})$, denoted by $N_{R(j)}$ and $N_{Q(i)}$, with interpolated data vectors, $D_{R(j)}$ and $D_{Q(i)}$, respectively. Top-left: schematic of our MML framework, extending **Fig. 2** (top-left). We define two hypotheses for R_j and Q_i: Hypothesis **A:** time points match, and Hypothesis Φ: time points mismatch. Next we compute the total (per-datum) encoding length of **A** and D, and the total (per-datum) encoding length of Φ and D. We define $Cost_{match}(i, j)$ as the difference between them, measured in nits (unit of Shannon information). **b,** Example cases of distributional differences (due to differences either in mean or variance, or in both) between $R(j)$ and $Q(i)$, and their $Cost_{match}(i, j)$. **c,** How $Cost_{match}(i, j)$ increases when differences increase. Left-plot: $Cost_{match}(i, j)$ between standard Gaussian distribution $N(0, 1)$ and $N(\mu, 1)$ for $\mu \in [0, 9]$ at 50 equispaced points. 5000 data points were randomly-sampled from each $N(\mu, 1)$ distribution to represent itself. Right-plot: $Cost_{match}(i, j)$ between $N(0, 1)$ and $N(0, \sigma)$ Gaussian distributions for $\sigma \in [0.1, 3]$ at 50 equispaced points. 5000 data points have been randomly sampled from each $N(0, \sigma)$ distribution to represent itself. Illustration in **a** was created using BioRender (https://biorender.com).

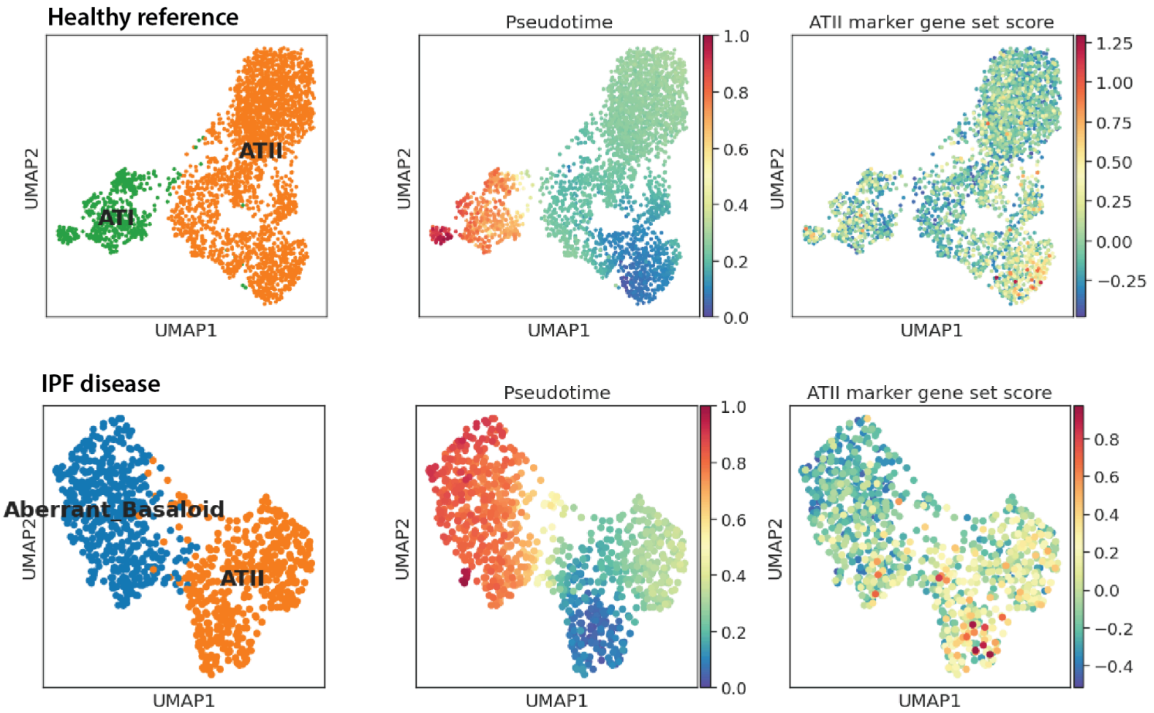

Supplementary Fig. 2 | Healthy versus Idiopathic Pulmonary Fibrosis (IPF) disease case study. The differentiation trajectories of alveolar type 2 (AT2) cells into alveolar type 1 (AT1) cells in the healthy lung aberrant basaloid cells (ABC) in the IPF lung, their estimated Diffusion Pseudotime, and the gene set score of the AT2 progenitor marker genes (*AXIN2, FGFR2, ID2, FZD6, LRP5, LRP6*) on the UMAP projection of (top) healthy and (bottom) IPF cells in the Adams et al. (2020)[1] dataset.

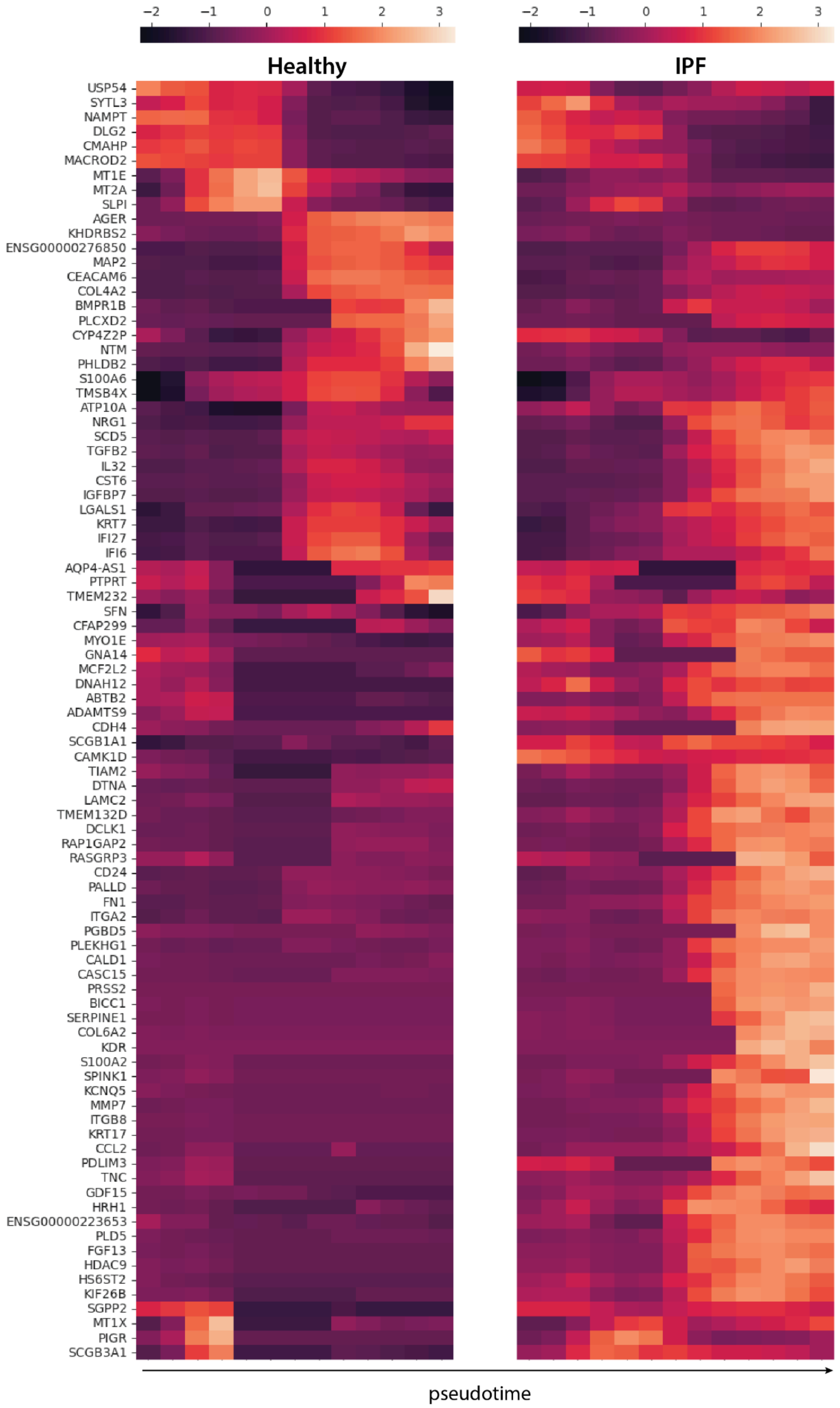

Supplementary Fig. 3 | Healthy vs IPF case study: Heatmap of the smoothened (interpolated) and z-normalized mean log1p (i.e. per-cell total sum of the raw transcript counts normalized to 10,000 and log1p transformed) gene expression of 88 marker genes of

aberrant basaloid cells (ABC). All interpolations were generated using our Genes2Genes framework.

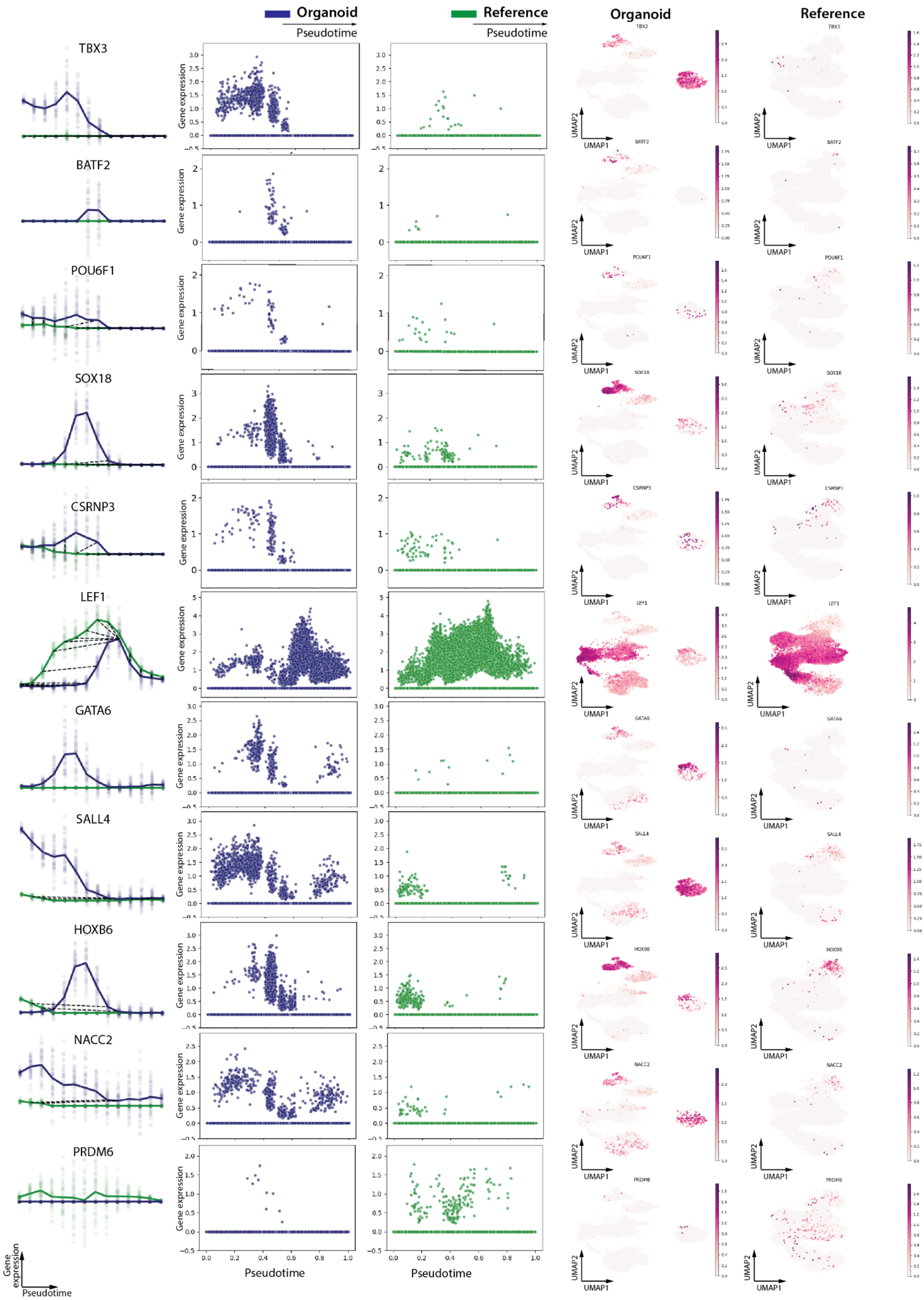

Supplementary Fig. 4 | Gene-level alignments of example genes in the Pan fetal reference vs. artificial thymic organoid alignment. These are identified under the clusters with interesting alignment patterns as illustrated in **Extended Data Fig. 9c**. Each row panel

presents data for a single gene. Left plot: the interpolated log1p normalized (i.e. per-cell total sum of the raw transcript counts normalized to 10,000 and log1p transformed) expression (*y*-axis) against pseudotime (*x*-axis). The bold lines represent mean expression trends, while the faded data points are 50 random samples from the estimated expression distribution at each time point. The black dashed lines represent matches and warps between time points. Middle two plots: the actual log1p normalized expression (*y*-axis) against pseudotime (*x*-axis). Each point represents a cell. Right two plots: The same UMAP visualization as in **Extended Data Fig. 6**, subsetted to *in vitro* cells from ATO in the left plot, and *in vivo* cells from the pan fetal reference, colored by the corresponding gene expression value.

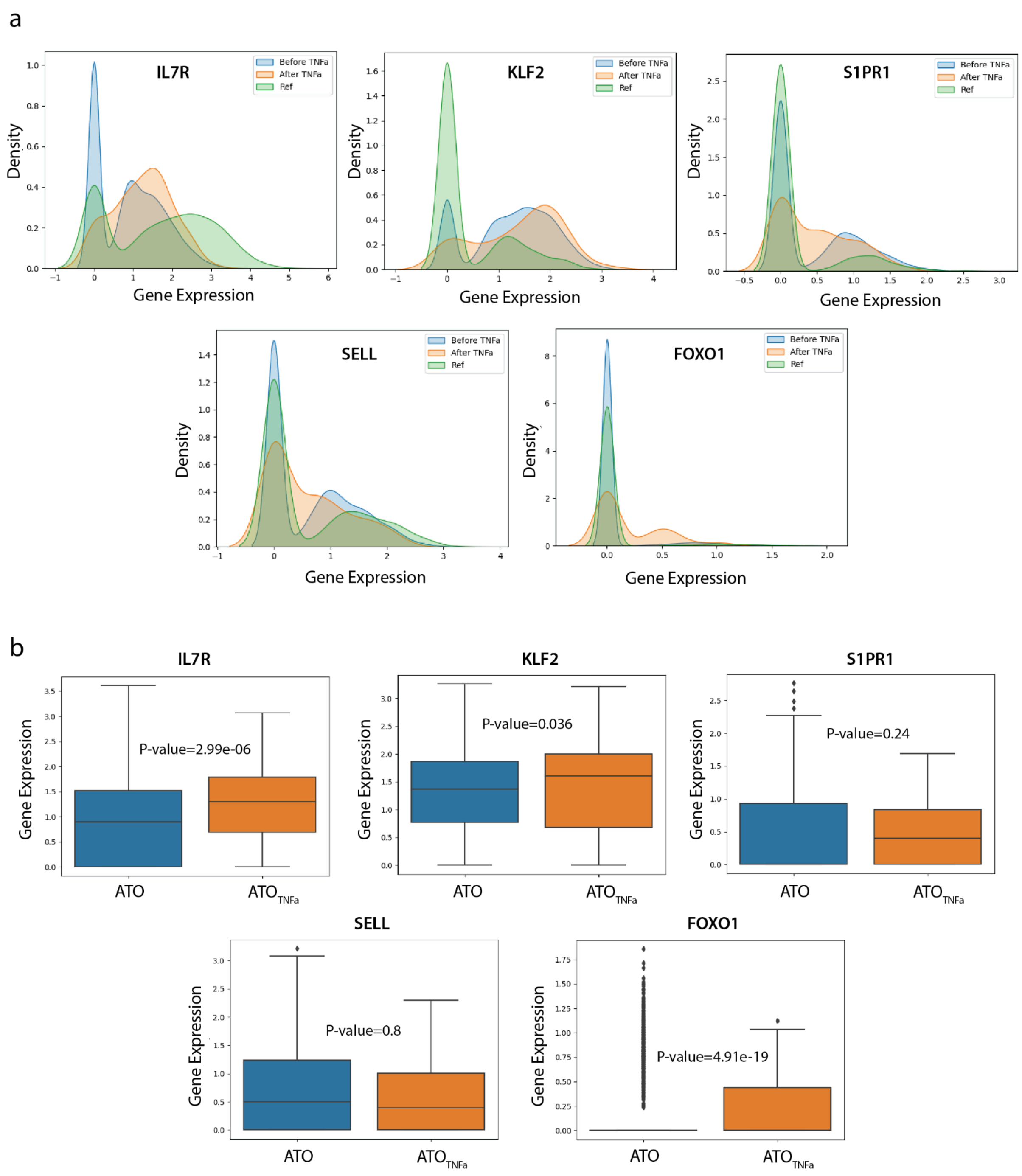

Supplementary Fig. 5 | Expression distributions of the marker genes of Single Positive T cell maturation. a, Density plots of the gene expression distributions of *IL7R, KLF2, S1PR1, SELL*, and *FOXO1* before and after TNFα treatment of the ATO compared to the pan fetal reference. **b,** Boxplot visualization of the gene expression distributions of the same marker genes, compared between the wild-type ATO and the TNFα treated ATO ($ATO_{TNFα}$), with P-values under the Man-Whitney U test (one-sided with alternative=‘less’) reporting the significance of the increase in expression level after the treatment. Each blue boxplot and orange boxplot show the distribution of n=6558 ATO SP T cells and $ATO_{TNFα}$ n=116 T cells, respectively. Boxplot visualizes the interquartile range (IQR) covering the 25% and 75%

quantiles, with line indicating median, and whiskers extending to 1.5 * IQR. Dots represent outlier cells.

a

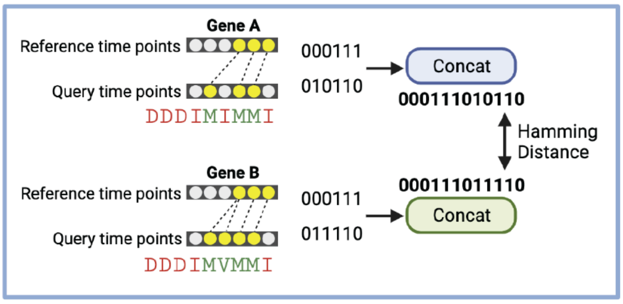

b **Hierarchical cluster diagnostics for Hamming distance across all datasets**

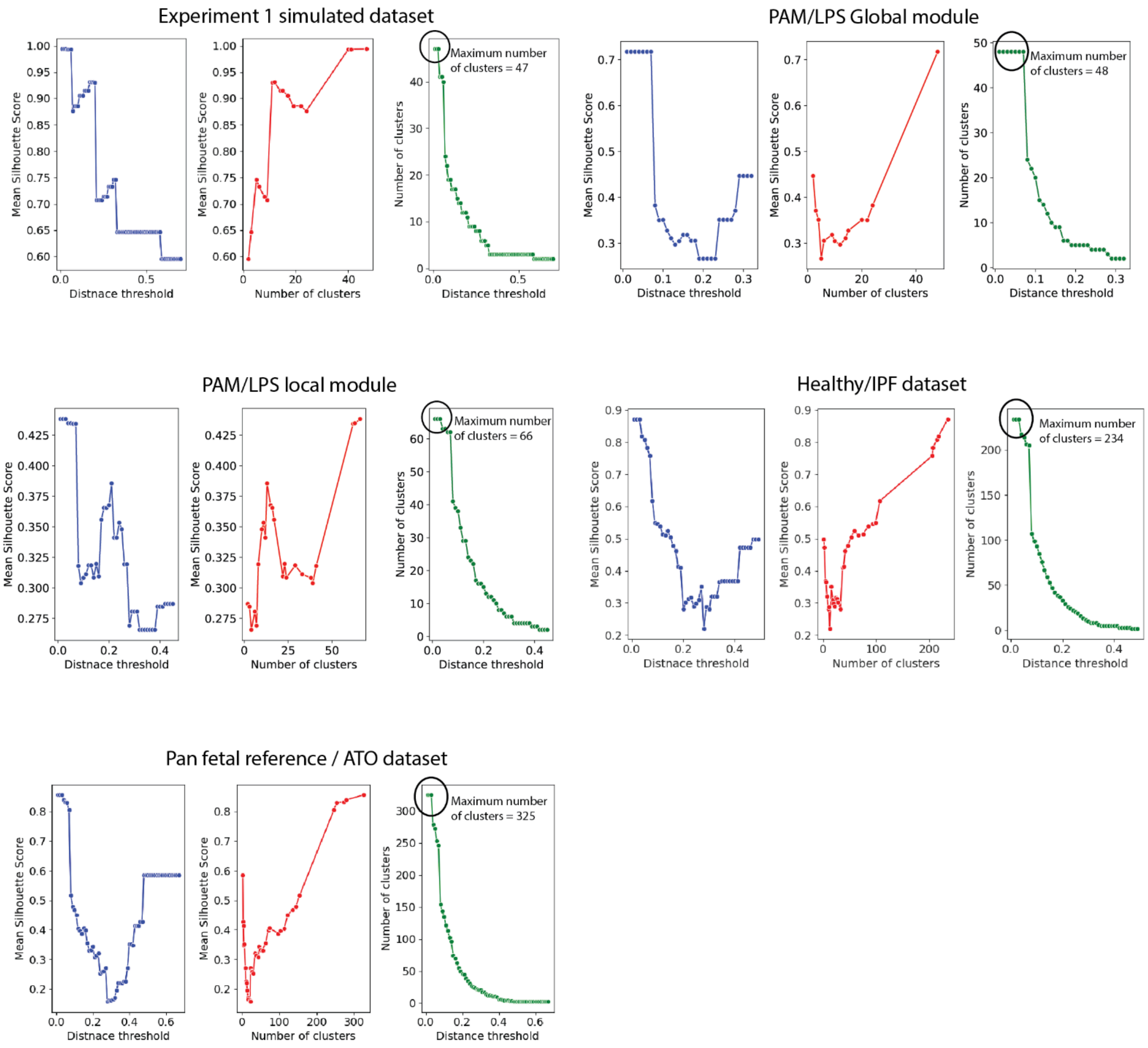

Supplementary Fig. 6 | Cluster diagnostic plots for Hamming distance. a, Schematic illustration of how the Hamming distance is computed between two 5-state alignment strings. Both 5-state strings are first binary-encoded independently to obtain two equal-length binary strings (as described in **Methods**), which are then used to compute the Hamming distance between them. **b,** Cluster diagnostic plots for the Hamming distance based hierarchical

agglomerative clustering of gene alignments across all the relevant datasets explored in the manuscript. These plots report the mean Silhouette score for the clustering structure when varying the Hamming distance threshold (or the number of clusters). Unlike when using Levenshtein distance, the highest number of clusters in these cases does not necessarily represent the number of all unique 5-state alignment strings. Illustration in **a** was created using BioRender (https://biorender.com).

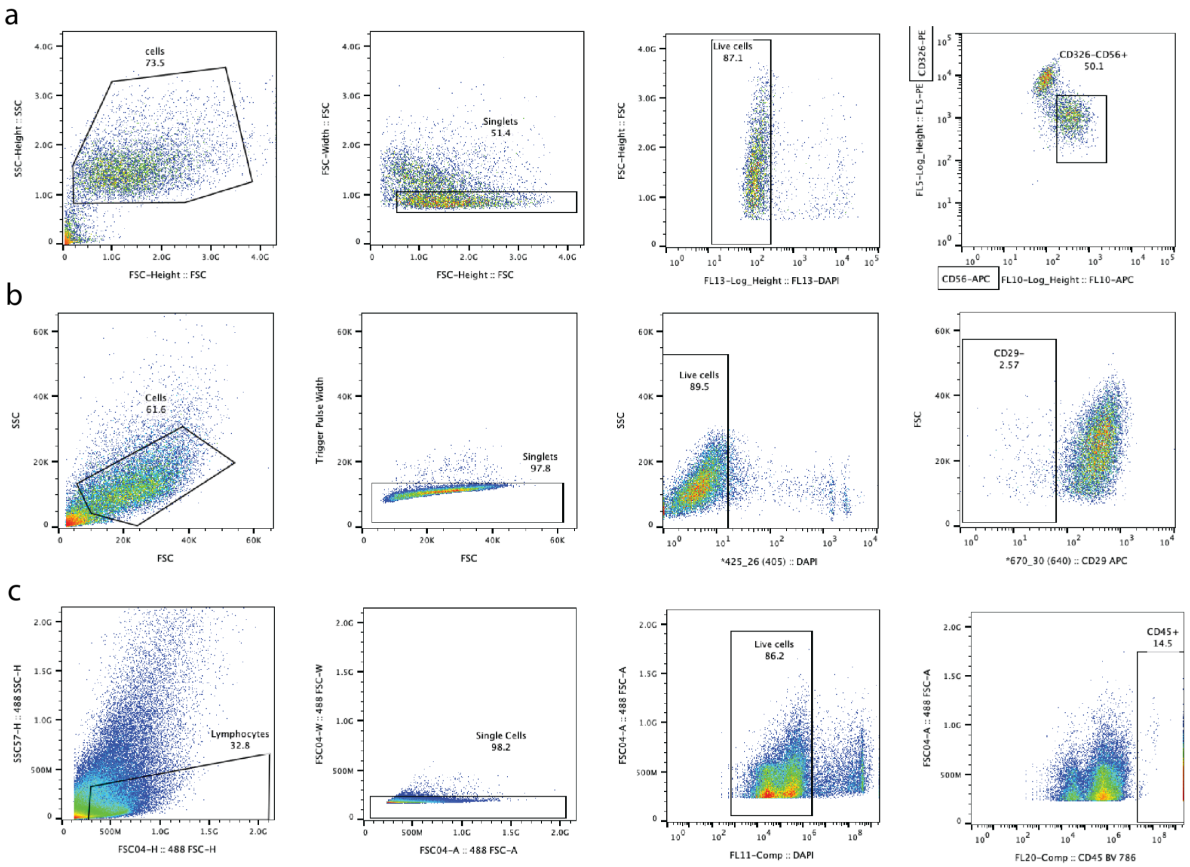

Supplementary Fig. 7 | Representative flow cytometry plots showing the sorting strategy for the ATO experiment. a, Representative FACS plots showing the sorting strategy on day−14. Human embryonic mesodermal progenitors were gated from live single cells which were $CD326^{-}CD56^{+}$. **b,** Representative FACS plots showing the sorting strategy on day −7, day 0 and week 3. Live human cells were gated from live single cells which were anti-mouse $CD29^{-}$. **c,** Representative FACS plots showing the sorting strategy for TNFα-treated ATO at week 7. Single cells were sorted for $DAPI^{-}CD45^{+}$ cells before 10X loading.

Supplementary Tables

Framework component	CellAlign[2]	TrAGEDy[3]	Genes2Genes
Input	(1) Log-normalized single-cell gene expression data<br>(2) Pseudotime estimates of the cells inferred using any available method of choice		
Algorithm	Uses Dynamic Time Warping (DTW) algorithm.	Builds on top of CellAlign[2], and performs post-hoc changes to the DTW alignment to capture mismatches.	Combines DTW and Gotoh's biological sequence alignment[4] through a new dynamic programming algorithm.
Alignment states	Handles matches and warps only, subjected to a weight scheme with constant weight for warp open/extension	Identifies optimal start and end time points of the trajectories for DTW alignment to exclude regions of mismatch at the beginning and end. It further filters the DTW aligned regions based on alignment cost thresholding to identify mismatches.	Handles matches, warps, and mismatches jointly, subjected to a five-state machine with state transition probabilities, handling gap/warp open/extension.
Trajectory Interpolation	Interpolates data using a Gaussian kernel-based weighted mean expression.	Extends CellAlign[2] interpolation to use a cell density weighted window size.	Extends CellAlign[2] interpolation to distributional interpolation using weighted variance.
Distance measure between a pair of reference and query time points	Uses min-max normalized, mean gene expression based Euclidean distance measure, to identify similar trends of expression dynamics across two conditions.	Uses Spearman correlation, which does not require gene expression scaling as done in CellAlign[2], but does not support gene-level alignment.	Uses a minimum message length inference based distributional distance measure, aiming to compare the gene expression distributions between two conditions.
Alignment output	Outputs only a single, high-dimensional alignment (across a given gene list). A single, gene-level alignment can be obtained by giving a single gene input.	Modified, high-dimensional DTW alignment after pruning the matches. A single, gene-level alignment can be obtained by giving a single gene input.	Outputs gene-specific alignments with an explicit alignment state description via a five-state alignment string for all the given genes. Can output an aggregate alignment for any given set of genes.
Alignment	Can cluster genes only	Does not explicitly	Can cluster genes based

clustering	based on the pseudotime shifts in their alignments.	discuss clustering.	on their five-state alignment strings, covering both matches and mismatches.
Differential expression capture	Requires additional downstream tasks to extract differential genes and regions (e.g. local DTW alignment with user-defined similarity threshold).	Sliding window soft clustering approach to extract DE using t-test/Mann-Whitney U test.	A gene-specific alignment output itself is a direct description of the differential expression status along the time axis. Provides a ranked list of genes based on their alignment similarity percentage.

Supplementary Table 1 | List of features included in trajectory alignment frameworks.
A table outlining and comparing the features of CellAlign[2], TrAGEDy[3] and G2G.

Supplementary Table 2
Grid search results for optimal 5-state machine parameters

Supplementary Table 3
Over-representation analysis results of healthy vs. IPF (under <=40% alignment similarity threshold)

Supplementary Table 4
Over-representation analysis results of T1 vs. ATO (under <=40% alignment similarity threshold)

Supplementary Table 5
Over-representation analysis results of T1 vs. ATO pluripotent genes cluster

Supplementary Table 6
Over-representation analysis results of T1 vs. ATO DN onwards (under <=40% alignment similarity threshold)

Supplementary Table 7
Over-representation analysis results of CD8+T vs. ATO DN onwards (under <=40% alignment similarity threshold)

Supplementary Table 8
ATO metadata
ATO_manifest.csv

Supplementary Table 9
ATO CITE-seq metadata
ATO_hashtagging.csv

Supplementary Table 10

Expression of selected TNFα pathway and marker genes in fetal thymus Visium data.
Visium_TNFa_expression.csv

Supplementary Data (zip file)

- ***SimulationExperiment1***
  - *TrAGEDy*
    - *TrAGEDy_5state_strings_minimumcut.csv*
    - *TrAGEDy_5state_strings_nullcuttype.csv*
    - *TrAGEDy_5state_strings_group_wise_[euclidean_nullcut].csv*
    - *TrAGEDy_5state_strings_group_wise_[euclidean_minimum].csv*
  - *CellAlign*
    - *cellalign_3state_strings.csv*
    - *cellalign_3state_strings_group_wise.csv*
  - *G2G*
    - *G2G_SimulationExperiment1_alignments.csv*

- ***SimulationExperiment2***
  - *TrAGEDy*
    - *TrAGEDy_mincut*
      - *<perturbation_type>_size<perturbation_size>.csv*
    - *TrAGEDy_nullcut*
      - *<perturbation_type>_size<perturbation_size>.csv*
  - *G2G*
    - *<perturbation_type>_size<perturbation_size>gene_alignments.csv*

- ***G2G_negative_control_alignments.csv***
- ***G2G_PAMLPS_global_module_alignments.csv***
- ***G2G_PAMLPS_local_module_alignments.csv***
- ***G2G_HealthyIPF_alignments.csv***
- ***G2G_RefATO_alignments.csv***
- ***G2G_RefATO_SPT_DNonwards_alignments.csv***
- ***G2G_RefATO_CD8_DNonwards_alignments.csv***

- ***SupplementaryFigures***

Supplementary Note

Additional analysis for *in vivo*, *in vitro* T cell comparison

We performed G2G alignment using *in vivo* conventional CD8+T cells and the relevant T lineage precursors (DN T cells onwards), with the results shown in **Extended Data Fig. 10b** . The most significantly enriched gene set among the mismatched genes for both cases remains the same (i.e. TNFα signaling via NF-κB pathway). To further explore differences in the two alignment results, we focused on genes that showed the most dissimilar alignment results (genes that had alignment similarity differences > 0.5 or < -0.5 at the last stages of the trajectories) (**Extended Data Fig. 10c**). For this, we looked at the difference of alignment similarity percentage in the last six alignment states of the 5-state gene-level alignment strings, and the $\log_2$ fold change of the mean interpolated gene expression in the last time bins, as heuristics to understand their differences.

Three of the genes, *SOX4, FOXP1* and *ARID5B* had large $\log_2$ fold change differences (absolute $\log_2$ fold change > 1) between type 1 innate T cells and CD8+T cells. For these three genes, the expression dynamics of *in vitro* T cell development are more similar to those of *in vivo* type 1 innate T cells, whereas *in vivo* CD8+T cells had higher *SOX4*, *FOXP1* and lower *ARID5B* expression in the last stages of development (**Extended Data Fig. 10c**). While the role of *SOX4* in CD8+T cell development is unclear, *FOXP1* has been shown to maintain a quiescent profile in naive CD8+T cells[5,6] , and our results are in keeping with a more activated profile in type 1 innate T cells. *ARID5B* has been reported to regulate metabolic programming and promote IFNγ production in NK cells[7]. The higher expression in type 1 innate T cells might explain some of their NK-like features[8,9]. We also note *LEF1* which shows a moderate alignment similarity difference and high $\log_2$ fold change; a gene that is known to be highly expressed in CD8+T cells compared to the type 1 innate T cells. On the other hand, for *BHLHE40*, which is downstream of TNFα signaling and other pro-inflammatory cytokines[10], its expression dynamic in *in vitro* T cell development is more similar to that in CD8+T cells, while the *in vivo* type 1 innate T cells have increased expression at the end. [10]

Spatial variation in TNFα pathway genes in thymus

To validate the increase in TNFα pathway genes we observed in the last stage of *in vivo* T cell development is not caused by *ex vivo* stress or handling artifacts during single cell dissociation from tissue, we followed the recommendation from Marsh et al. 2022[11] and investigated orthogonal *in situ* methods such as spatial transcriptomics. We took the Visium spatial transcriptomic data of 3 fetal thymic slides from our developing human immune atlas[9] and compared the TNFα pathway gene expressions between cortex (where T cell progenitors are) and medulla (where mature T cells are) regions. The annotation of tissue regions on Visium spots were inferred by clustering of H&E image features[9]. We selected the TFs in TNFα pathway that showed increased expression at the end of the *in vivo* T cell development but no increase in *in vitro* T cell development in **Fig. 6d**, together with mature T cell marker

CD27 and T cell progenitor markers *RAG1* and *RAG2*. Results are shown in **Supplementary Table 10** (with P-values computed using the two-sided Wilcoxon rank-sum test). TFs in TNFα pathway have higher expression in the medulla than cortex, which is consistent across all 3 slides, corroborating with what we have observed in the scRNA-seq data with the increase in expression at the end of *in vivo* T cell development. As expected, *CD27* expression is higher in medulla than cortex, whereas *RAG1* and *RAG2* have higher expression in cortex than medulla.

TNFα validation experiment results

This section presents results for all statistical tests that check the MML distances in gene expression distributions of the SP T cells in the ATO and the type 1 innate T cells in the pan fetal reference, before and after TNFα treatment. Across all TFs and different relevant pathway gene sets (i.e. TNFα pathway, P38, JNK, NF-ϰB canonical signaling known to be targeted by TNFα signaling), we tested the mean change in MML distances of gene expression across all significantly distant genes, as well as across all genes.

The significance of distance in gene expression between reference and query was computed under the empirical CDF null model. When considering all genes, the mean change statistic may be misleading due to the noise in distances. Checking between the significant distances allows us to avoid such bias. Overall, we see that on average, the significantly changed genes have decreased in their gene expression distance.

**Gene set**	**Significance level of differential expression**	**# of significantly distant genes diffset$_{UNTREATED}$**	**# of significantly distant genes diffset$_{TREATED}$**	**Mean distance of diffset$_{UNTREATED}$ (nits)**	**Mean distance of diffset$_{TREATED}$ (nits)**	**P-value for change in distance (Mann-Whitney U test)**	**Mean distance change of all genes (nits)**
All TFs (1371)	0.005	12	35	31.51	12.91	0.0006131978745461366	0.404 → 0.6 (-)
All TFs (1371)	0.05	24	91	18.54	6.67	2.9617483396576014e-05	
TNFα signaling via NF-ϰB - TFs (42)	0.005	11	13	28.54	9.98	0.011925364427524157	7.77 → 3.36 (+)
TNFα signaling via NF-ϰB - TFs (42)	0.05	15	19	21.38	7.26	0.04460916201610702	
TNFα signaling via	0.005	32	30	21.18	10.41	0.0084802992372226	3.771 →1.899

NF-ϰB - all genes (196)						36	(+)
TNFα signaling via NF-ϰB - all genes (196)	0.05	57	54	12.6	6.5	0.1558075120839859	
All TFs excluding TNFα TFs (1329)	0.005	3	27	24.84	12.47	0.036206896551724134	0.171 → 0.512 (-)
All TFs excluding TNFα TFs (1329)	0.05	12	70	10.65	6.6	0.014467541263839772	
NF-ϰB canonical pathway (215)	0.005	8	29	24.45	10.76	0.0003306048846845811	1.199 → 1.858 (-)
NF-ϰB canonical pathway (215)	0.05	18	77	12.48	5.01	0.0007696929346291883	
P38 (50)	0.005	4	6	40.5	12.23	0.01904761904761905	3.361 → 1.795 (+)
P38 (50)	0.05	4	12	40.5	6.94	0.002197802197802198	
Jnk(155)	0.005	2	15	16.03	8.85	0.08823529411764706	0.525 → 1.307 (-)
Jnk (155)	0.05	13	51	5.04	3.77	0.02967266623081871	

We note that for many cases, the distance has significantly dropped despite the increase in the number of genes with significant distances after TNFα treatment. This is because the highly distant genes before treatment have now got closer, outweighing the increase in the number of differential genes.

For TFs, there are 20 TFs that are significantly distant both before and after treatment, implying 4 TFs (*FOSB, GPBP1, KLF6, REL*) have rectified its distance after treatment. Among the 20 common TFs, 14 TFs (70%) are on the path to getting closer to the reference (i.e. *CEBPD, CREM, CSRNP1, FOS, JUN, JUNB, NME2, NR4A1, NR4A2, PA2G4, TSC22D1, YBX1, ZNF331, ZNF683*). There are 71 TFs that got significantly different after treatment, out of which, 11 TFs (i.e. *BHLHE40, HIF1A, JUND, KDM2A, NCOA3, NFKB2,*

RELB, RUNX3, STAT4, USF2, ZBTB1) surpassed the reference expression in the right direction (high expression or low expression), suggesting the need for some modulation.

For genes in the TNFα signaling pathway, the number of significantly distant genes have dropped from 57 to 54 after TNFα treatment. There are 22 genes that have rectified its distance after treatment (*ATF3,BIRC2,CCL4,CCNL1,CD69,DUSP1,FOSB,FOSL2, ID2,IER2,KDM6B,KLF6,MAP3K8,NFE2L2,PDE4B,PTGER4,SGK1,SIK1,SLC2A3,SQSTM1, TNFAIP3,ZC3H12A*). 35 genes remained distant after treatment, out of which, 24 genes got closer to the reference than before (i.e. *BTG2, CEBPD, DNAJB4, DUSP2, DUSP4, FOS, GADD45B, GEM, GPR183, IER3, IL7R, JUN, JUNB, MXD1, NFKBIA, NR4A1, NR4A2, PHLDA1, PPP1R15A, REL, RHOB, SAT1, TSC22D1, ZFP36*). There are only 19 genes that were similar before, but got distant after treatment, out of which 5 genes (*BHLHE40, DUSP5, IER5, NFKB2, RELB*) surpassed the reference expression in the right direction.

We note that other TFs and genes could become more distant after TNFα treatment as a result of TNFα induced stress response. This may explain why the average distances across all genes in the NF-ϰB and JNK pathways have increased (as stress seems to be activating those pathways – References: https://www.ncbi.nlm.nih.gov/pmc/articles/PMC2823860/ , https://www.ncbi.nlm.nih.gov/pmc/articles/PMC5046695/). However, despite such changes, the fact that CellTypist still annotates the TNFα treated ATO cells as type 1 innate T, and their increased expression of gene markers of SP T cell maturation suggest more maturity. Thus we conclude that TNFα signaling is a potential direction for the ATO protocol refinement, which is worth exploring further.

Inspecting the known markers of SP T cell maturity
We also checked the differences in the expression distributions of known SP T cell maturation markers reported in literature. *IL7R* has shown to be initiated in mature SP thymocytes, and its expression is dependent on NF-ϰB signaling, triggered by TNFα signaling[12,13,14].

***Comparing the MML distances between ATO and reference before and after TNFα treatment* (Supplementary Fig. 6a):** The MML distance of *IL7R* between the reference and $ATO_{TNF\alpha}$ is 13.347 nits, which is significantly different (P-value=~0 under empirical CDF), while it significantly drops to 4.041 nits after TNFα treatment, with an increased induction of expression as expected from mature SP T cells. It remains different between reference and $ATO_{TNF\alpha}$, however with a higher p-value than before (P-value=~0.0004 under empirical CDF). *KLF2* is more upregulated in the ATO than the reference, and has further upregulated in $ATO_{TNF\alpha}$, thus increasing the distance from 17.0983 nits to 21.9164 nits. *KLF2* upregulation is expected in mature SP T cells[15]. *S1PR1* becomes less distant between reference and $ATO_{TNF\alpha}$ (from 1.3549 nits to 1.2962 nits) even if it remains differential at 0.01 significance. *FOXO1* on the other hand is more upregulated in $ATO_{TNF\alpha}$ than before, increasing the distance to the reference. *SELL* expression is known to be maintained by

FOXO1 and *KLF2* [16]. However, *SELL* shows no significant difference to the reference before and after TNFα treatment.

***Complementary comparison of marker expression between ATO and ATO$_{TNF\alpha}$* (Supplementary Fig. 6b):** Complementary to the above analysis, we performed the Mann-Whitney U test to check if the ATO expression is significantly lesser than ATO$_{TNF\alpha}$ expression for the above marker genes. Accordingly, *IL7R, KLF2, FOXO1* have significantly increased expression, while *S1PR1* and *SELL* do not show significant difference before and after treatment.

Trajectory alignment across different pseudotime estimations

G2G can be used to align pseudotime trajectories estimated using any of the many pseudotime inference methods available today (e.g. Diffusion pseudotime[17], Palantir[18], GPLVM[19], Monocle[20], Slingshot[21] etc.). However the trajectory alignment of datasets is heavily driven by the choices made a priori for their trajectory inference, and therefore it is important that the user initially verifies the reliability of their input trajectories, and validate the information gained from final gene-level alignments (experimentally, functionally and/or based on current literature) before coming to biological conclusions.

There is a massive space of variables involved in a trajectory inference. For instance, using different low-dimensional embeddings of the data, different gene (feature) selection strategies, or different integration strategies including different combinations of variables for batch correction, may give different pseudotime distributions. Different pseudotime inference approaches such as graph based methods (e.g. minimum spanning trees) and Markov model based methods (also under different hyperparameter settings), can output different pseudotime distributions. Further, depending on the research question the user is interested in, the user may integrate either the entire dataset or different subsets and combinations of lineages and cell types together, which will also govern the final pseudotime estimation. The resolution of pseudotime will be based on the level of such integration. All these choices control the shape, skewness, range, and the resolution of pseudotime, and the resultant cell-type compositions across pseudotime, which ultimately controls the matches and mismatches captured by pseudotime trajectory alignment.

Inaccurate and unreliable choices made in trajectory inferences result in inaccurate and unreliable trajectory alignments as well. Therefore we recommend users to evaluate the accuracy of their trajectories before performing any sort of comparative analysis. It is indeed challenging to evaluate the accuracy of a resultant trajectory as we do not know the absolute time of each cell, and also impractical to benchmark pseudotime across all the different choices and practices as we do not know the ground truth. Thus it is still an open area of research. However, users could always go for a reasonable pseudotime distribution as long as it fairly represents the expected cell-type compositions along the entire trajectory. Users could refer to benchmark studies in literature such as the comprehensive review paper by

Saelens et al (2019)[21,22] and explore several trajectory inference approaches before deciding on the final pseudotime trajectories to align. Users can also use methods that allow the incorporation of time priors (e.g. the GPLVM approach we used for ATO pseudotime estimation based on the real sampling time points of the cells) to obtain reliable estimates.

To test the robustness of results from G2G trajectory alignment across the differences in the pseudotime estimates, we explored the tools: Monocle3, Slingshot, Palantir, and Diffusion pseudotime (DPT) (in addition to the GPLVM estimation reported in the main text) on our pan fetal reference and ATO datasets (for trajectories starting from the early DN stage onwards). Apart from GPLVM, only Monocle3 gave a reliable and reasonable pseudotime estimation for these datasets, with the expected cell type compositions fairly represented along pseudotime. (See **Figure S1** for the pseudotime density plots and cell type composition plots along time across all the pseudotime estimators). Accordingly, we re-performed G2G alignment using Monocle3 estimates and found that the TNFα pathway remains the one under which the most of the TFs are significantly different with an end mismatch, consistent with our results from using GPLVM based pseudotime estimates. Nevertheless, we take GPLVM estimates to be more reliable in this context, because unlike Monocle3, GPLVM enabled us to incorporate time priors based on the real sampling time points of the ATO. Further, inspecting the cell density across pseudotime, GPLVM gives more evenly distributed estimates than the estimates from Monocle3 (**Figure S1**).

We also re-estimated pseudotime for healthy/IPF datasets using Monocle3, Slingshot and Palantir (in addition to DPT estimation reported in the main text) which gave expected cell type compositions fairly represented along pseudotime (See **Figure S2**). We then re-performed G2G alignment across all tools, resulting in gene-level alignments for which the most genes are significantly different under the Epithelial Mesenchymal Transition pathway (as the top hit in the over-representation analysis), consistent with our results from using DPT estimates. (Note: We did not compare GPLVM for this, since there are no time priors available for the healthy/IPF datasets).

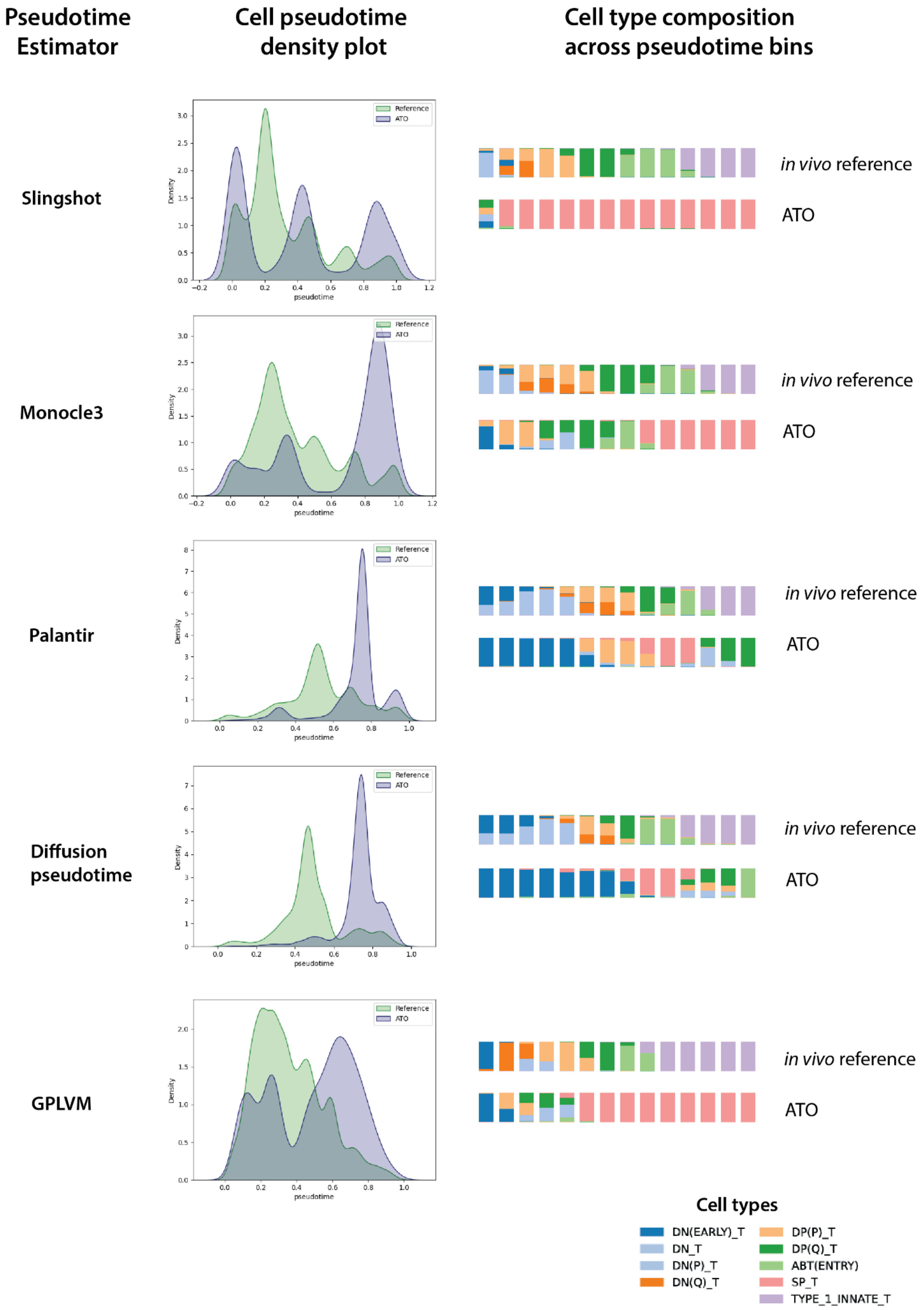

Figure S1: Pseudotime density plots and cell type composition plots along pseudotime across pseudotime estimators: Slingshot, Monocle3, Palantir, Diffusion pseudotime, and GPLVM,

on the pan fetal reference and ATO datasets which include T cell differentiation trajectories from DN stage onwards.

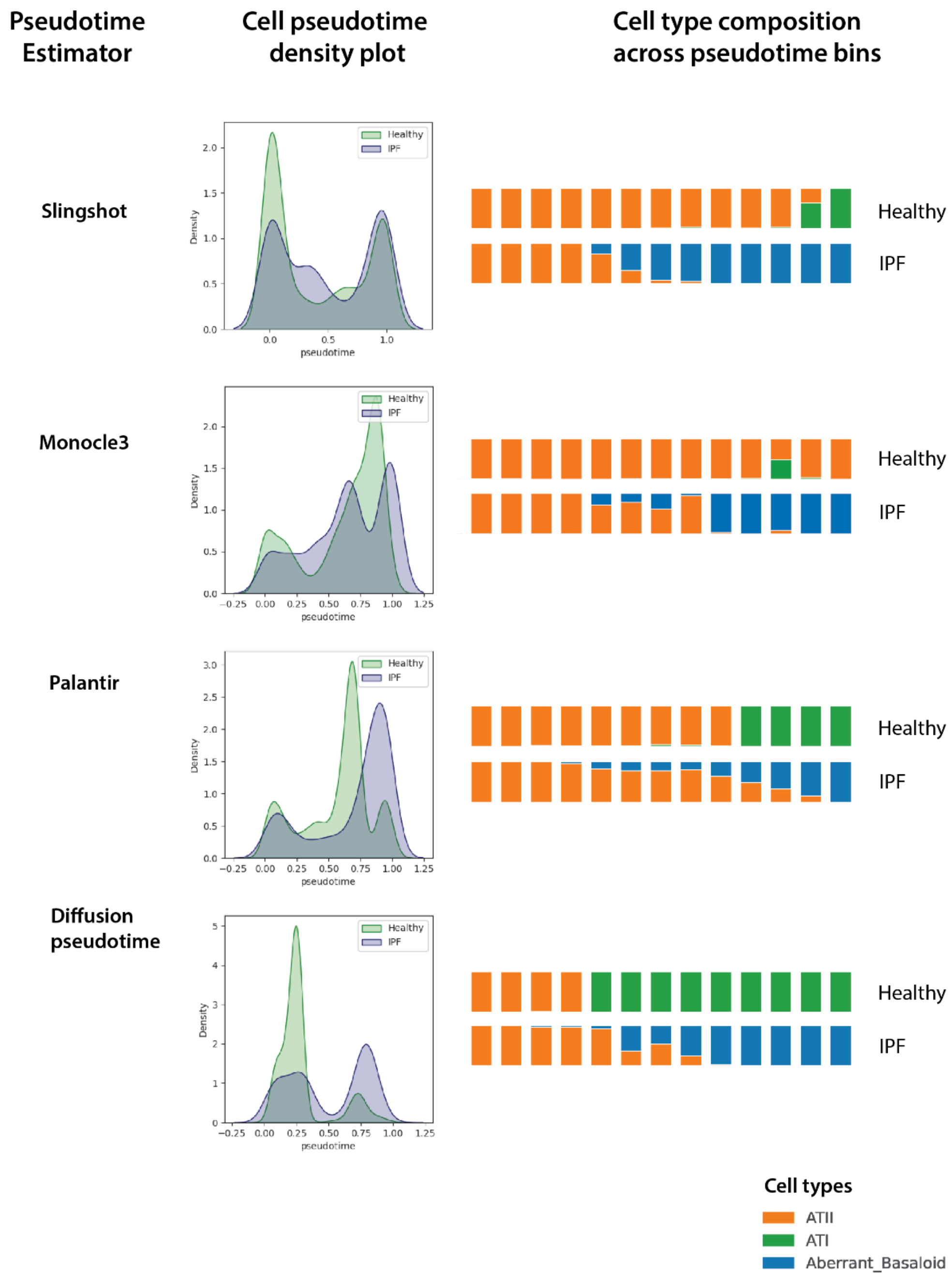

Figure S2: Pseudotime density plots and cell type composition plots along pseudotime across pseudotime estimators: Slingshot, Monocle3, Palantir, and Diffusion pseudotime, on the healthy and IPF datasets.

Trajectory alignment using low-dimensional, latent embeddings

G2G currently aligns each specified gene independently, thus requiring a clustering of gene-level alignment strings to identify gene groups with similar alignment patterns. As noted in the Discussion, we can infer aggregate alignments for a priori known gene modules (e.g. biological pathway gene sets or regulons). In addition, we can also align dimensions of a low dimensional, latent embedding of the reference and query datasets rather than their actual gene dimensions. For instance, a user could input data to G2G after steps such as batch correction and dimensionality reduction, i.e., latent variables from an scVI embedding or principal components (PCs) from principal component analysis.

To test this, we aligned the 10 scVI latent dimensions of the pan fetal reference and ATO data using G2G in the same way as performed for the 1371 TFs in our reported study. The average alignment (IIIDIDIDMMMMMMMMMDD) gives early mismatches and late mismatches consistent with the average alignment reported in the main text. Similar result (IIIDMMMMMMMMMMMMDD) was observed when aligning the first 50 PC components of the reference and query datasets. Separately, testing the PC component alignment of healthy/IPF datasets, G2G again outputs an average alignment (MMMMMMIIIIDDMMMDD) of early matches and late mismatches consistent with the results reported in the main text.

Overall, the aggregated results from latent dimension alignment could act as cell-level alignment, and such a low-dimensional alignment process is more time-efficient than the current gene-level approach. However, the alignment result from each PC or latent variable might be difficult to interpret biologically, thus requiring further research to identify genes that contribute to the alignment pattern of each latent dimension.

Alignment in the presence of batch effects

A key feature of G2G is that it models gene expression at a particular time point t in a trajectory as a Gaussian distribution, taking into account both mean and variance of gene expression as weighted estimates based on all the cells in the trajectory. (i.e. the cells in time points closer to t have greater contributions towards the estimation, whereas cells in far away time points have lesser — and if they are too far, almost negligible – contributions towards the estimation). Thus, for a given particular trajectory dataset (which represents either a reference or query system), the estimated gene expression distribution corresponding to time point t represents the variance caused by unknown batch effects and other confounders (donor-to-donor variability, technology, sample etc.). When there is a small batch effect, we expect low variance in the estimated Gaussian which mostly represents the natural variance of RNA expression amongst the similar type of cells. If there are large batch/confounder effects, then we expect a much higher variance in the estimated Gaussian.

Modeling gene expression as distributions enables G2G to account for the batch effects within the same system (either reference or query). However, G2G does not model or correct

for batch effects between the reference and query systems. Batch correction may overcorrect and obscure true biological differences, thus the approach of identifying all mismatches and investigating them in downstream analysis is a much safer choice. However if a user prefers a particular batch correction method, they could input data batch corrected (or confounders regressed) between reference and query, and then perform G2G alignment. Particularly, as described in the previous section, we can perform a G2G alignment between the batch-corrected latent dimensional embeddings (generated using a tool such as scVI) of the reference and query datasets.

Stability test to assess robustness of G2G alignment to individual-to-individual variability in trajectories

To test how the G2G method is affected by batch-to-batch variability in trajectories, we performed a stability test considering the individual-to-individual (donor) variability in the healthy/IPF and *in vitro/in vivo* T cell trajectories.

Stability test on healthy/IPF datasets: We performed a leave-50%-donors-out stability test on the healthy and IPF datasets. There were 28 donors and 31 donors in the healthy and IPF datasets, respectively. For the random sampling experiment, we considered only the 12 healthy donors and 7 IPF donors who had sufficient data (i.e. >=50 cells in each donor batch). We then randomly sampled 50% donors for 5 times, independently for healthy and IPF, creating 10 random donor subsets (including both, the sampled 5 subsets as well as their complementary subsets). Next we performed G2G alignment for all 10 donor subset pairs between healthy and IPF under the same setting of parameters and 994 genes we had for our previous alignment reported in the manuscript main text. We observed that the overall alignment pattern of early match and late mismatch which we found from our previously reported alignment is consistent across all these new 10 alignments as well (as shown below).

Iteration	Average alignment
1	MMMMMMMMMMIIDDDI
2	MMMMIDMMMMMMMDI
3	MMMMMMMMMIDIDMDI
4	MMMIVDDMMMMMMDIID
5	MMMMMMMMMWWMDIII
6	MMMMIDMMMMVMIDIDD
7	MMIMVDMMMMVIDDDIDID
8	MMMMMMMMMIDMMID
9	MMMMIDMMMMVMIDIDD
10	MMMMMMMMVIDDDMDII

We also repeated the healthy vs IPF G2G alignment for 10 iterations by downsampling each dataset (i.e. by 50% subsampling of cells) and again observed that the overall alignment pattern of early match and late mismatch is consistent (as shown below).

Iteration **Average alignment**

1	MMMMMMMMMMIIDDDI
2	MMMMMMMMMMIDDDII
3	MMMMMMMMMMIDDDII
4	MMMMMMMMMMMMID
5	MMMMMMMMMMIIDDID
6	MMMMMMMMMMMIDID
7	MMMMMMMMMMMIDID
8	MMMMMMMMMMIIDDID
9	MMMMMMMMMMIDDIDI
10	MMMMMMMMMMIIDIDD

Stability test on in vitro/in vivo* T cell *datasets: Additionally, we applied the same donor subset sampling strategy to the pan fetal reference dataset where we had 22 donors with sufficient data (>=50 cells) out of 33 donors. With 10 random donor subsets (each having 50% donors = 11 random donors), each reference random donor subset was aligned against the complete ATO dataset (Note: ATO dataset does not contain donors, but just 2 cell lines, therefore it was not downsampled). We again observed that the overall alignment results across all the 10 alignment iterations (as shown below) are consistent with our previously reported alignment (i.e. early mismatches and late mismatches) of the complete dataset alignment.

Alignment across the 42 TFs in the TNFα pathway:

Iteration	Average alignment
1	IIIDIDIMMMMMMMMMDDD
2	IIIDMMMMMMMMMMMDD
3	IIIDMMMMMMMMMMMDD
4	IIIDIDIMMMMMMMMMDDD
5	IIIDIMMMMMMMMMMDDD
6	IIIDIDMMMMMMMMMIDDD
7	IIIDIDIMMMMMMMMMDDD
8	IIIDIMMMMMMMMMMDDD
9	IIIDIMMMMMMMMMMDDD
10	IIIDIDIMMMMMMMMMDDD

Alignment across all 1371 TFs:

Iteration	Average alignment
1	IIIDIDMMMMMMMMMMDD
2	IIDMMMMMMMMMMMMD
3	IIDMMMMMMMMMMMMD
4	IIIDDMMMMMMMMMMMD
5	IIIDDMMMMMMMMMMMD
6	IIIDMMMMMMMMMMMDD
7	IIIDDMMMMMMMMMMMD
8	IIDMMMMMMMMMMMMD
9	IIIDMMMMMMMMMMMDD
10	IIIDDMMMMMMMMMMMD

Based on the above described stability tests, we conclude that the G2G method was still able to produce consistent alignment results despite the changes in donor compositions, and the overall alignment results were not influenced by any outlier individual. A user could also perform a similar stability test across alignment using different random batch subsets, to ensure robustness of the final conclusions.

Note on aligning two batches from the same trajectory
Even though in theory we expect 100% matched trajectories between individual batches that represent the same system (different 10X channels, individuals etc), we cannot guarantee such alignment when aligning two such batch trajectories. This is because they may have genuine differences in their trajectory representations (cell-type compositions) and/or large batch effects caused by biological/technical confounding variables. Therefore it is difficult to benchmark batch-to-batch alignments without knowing the expected (true) matching and mismatching behaviors across pseudotime. However G2G could be a tool to identify such differences between batches and support evaluating batch effects.

References

1. Adams, T. S. *et al.* Single-cell RNA-seq reveals ectopic and aberrant lung-resident cell populations in idiopathic pulmonary fibrosis. *Sci Adv* 6, eaba1983 (2020).
2. Alpert, A., Moore, L. S., Dubovik, T. & Shen-Orr, S. S. Alignment of single-cell trajectories to compare cellular expression dynamics. *Nat. Methods* 15, 267–270 (2018).
3. Laidlaw, R. F., Briggs, E. M., Matthews, K. R., McCulloch, R. & Otto, T. D. TrAGEDy: Trajectory Alignment of Gene Expression Dynamics. Preprint at https://doi.org/10.1101/2022.12.21.521424.
4. Gotoh, O. An improved algorithm for matching biological sequences. *Journal of Molecular Biology* vol. 162 705–708 Preprint at https://doi.org/10.1016/0022-2836(82)90398-9 (1982).
5. Feng, X. *et al.* Foxp1 is an essential transcriptional regulator for the generation of quiescent naive T cells during thymocyte development. *Blood* 115, 510–518 (2010).
6. Kaminskiy, Y., Kuznetsova, V., Kudriaeva, A., Zmievskaya, E. & Bulatov, E. Neglected,

yet significant role of FOXP1 in T-cell quiescence, differentiation and exhaustion. *Front. Immunol.* 13, 971045 (2022).

7. Cichocki, F. *et al.* ARID5B regulates metabolic programming in human adaptive NK cells. *J. Exp. Med.* 215, 2379–2395 (2018).
8. Park, J.-E. *et al.* A cell atlas of human thymic development defines T cell repertoire formation. *Science* 367, (2020).
9. Suo, C. *et al.* Mapping the developing human immune system across organs. *Science* 376, eabo0510 (2022).
10. Cook, M. E., Jarjour, N. N., Lin, C.-C. & Edelson, B. T. Transcription Factor Bhlhe40 in Immunity and Autoimmunity. *Trends Immunol.* 41, 1023–1036 (2020).
11. Marsh, S. E. *et al.* Dissection of artifactual and confounding glial signatures by single-cell sequencing of mouse and human brain. *Nat. Neurosci.* 25, 306–316 (2022).
12. Webb, L. V. *et al.* Survival of Single Positive Thymocytes Depends upon Developmental Control of RIPK1 Kinase Signaling by the IKK Complex Independent of NF-κB. *Immunity* 50, 348–361.e4 (2019).
13. Carty, F. *et al.* IKK promotes naïve T cell survival by repressing RIPK1-dependent apoptosis and activating NF-κB. *Sci. Signal.* 16, eabo4094 (2023).
14. NF-κB signaling mediates homeostatic maturation of new T cells. *Paperpile* https://paperpile.com/app/p/5bf5450b-fe7e-0ecd-b47f-44e59aeb429d.
15. Postselection thymocyte maturation and emigration are independent of IL-7 and ERK5. *Paperpile* https://paperpile.com/app/p/2e39eee8-980f-0a93-a55e-fdeea79c62bc.
16. Trinité, B. *et al.* Suppression of Foxo1 activity and down-modulation of CD62L (L-selectin) in HIV-1 infected resting CD4 T cells. *PLoS One* 9, e110719 (2014).
17. Haghverdi, L., Büttner, M., Wolf, F. A., Buettner, F. & Theis, F. J. Diffusion pseudotime robustly reconstructs lineage branching. *Nat. Methods* 13, 845–848 (2016).

18. Setty, M. *et al.* Characterization of cell fate probabilities in single-cell data with Palantir. *Nat. Biotechnol.* 37, 451–460 (2019).

19. Titsias, M. & Lawrence, N. D. Bayesian Gaussian Process Latent Variable Model. in *Proceedings of the Thirteenth International Conference on Artificial Intelligence and Statistics* 844–851 (PMLR, 13--15 May 2010).

20. Cao, J. *et al.* The single-cell transcriptional landscape of mammalian organogenesis. *Nature* 566, 496–502 (2019).

21. Street, K. *et al.* Slingshot: cell lineage and pseudotime inference for single-cell transcriptomics. *BMC Genomics* 19, 1–16 (2018).

22. Saelens, W., Cannoodt, R., Todorov, H. & Saeys, Y. A comparison of single-cell trajectory inference methods. *Nat. Biotechnol.* 37, 547–554 (2019).

Table2-optimal_state_machine_params_grid_search_results

	Convergence_025	Convergence_05	Convergence_075	Divergence_025	Divergence_05	Divergence_075	AllMatch	mean_inaccuracy_rate	params
**6**	0.8	1.8	0.8	0.6	0.2	0.0	1.4	0.11428571428571400	[0.99, 0.1, 0.7]
**17**	0.6	1.0	2.4	0.4	0.0	0.0	1.4	0.11836734693877600	[0.99, 0.3, 0.3]
**12**	0.8	1.6	1.6	0.4	0.0	0.0	1.4	0.11836734693877600	[0.99, 0.2, 0.5]
**16**	0.6	1.0	2.4	0.4	0.0	0.0	1.6	0.12244897959183700	[0.99, 0.3, 0.2]
**11**	0.8	1.6	1.6	0.4	0.0	0.0	1.6	0.12244897959183700	[0.99, 0.2, 0.4]
**5**	0.8	1.6	1.8	0.4	0.0	0.0	1.6	0.12653061224489800	[0.99, 0.1, 0.6]
**15**	0.6	1.2	2.4	0.4	0.0	0.0	2.2	0.13877551020408200	[0.99, 0.3, 0.1]
**4**	0.6	1.0	2.6	0.4	0.0	0.2	2.2	0.14285714285714300	[0.99, 0.1, 0.5]
**10**	0.6	1.0	3.0	0.4	0.0	0.0	2.2	0.1469387755102040	[0.99, 0.2, 0.3]
**3**	0.6	1.6	5.2	0.4	0.4	0.2	2.2	0.2163265306122450	[0.99, 0.1, 0.4]
**21**	0.6	3.0	5.2	0.4	0.4	0.0	1.2	0.22040816326530600	[0.99, 0.4, 0.1]
**9**	0.6	2.2	8.2	0.4	0.4	0.2	2.2	0.2897959183673470	[0.99, 0.2, 0.2]
**44**	3.8	8.6	3.2	0.6	0.6	0.6	3.2	0.42040816326530600	[0.9, 0.1, 0.7]
**2**	0.6	4.8	11.6	0.6	0.6	0.2	2.8	0.4326530612244900	[0.99, 0.1, 0.3]
**43**	6.6	12.0	5.6	0.8	1.4	1.4	4.2	0.6530612244897960	[0.8, 0.1, 0.7]
**8**	0.6	6.6	30.0	0.6	0.6	0.0	2.6	0.8367346938775510	[0.99, 0.2, 0.1]
**42**	9.2	14.4	6.6	2.6	3.0	3.8	5.8	0.926530612244898	[0.7, 0.1, 0.7]
**1**	2.4	12.6	38.0	0.6	0.8	0.6	3.0	1.1836734693877600	[0.99, 0.1, 0.2]
**41**	14.6	17.6	7.8	5.2	7.6	8.6	8.8	1.4326530612244900	[0.6, 0.1, 0.7]
**13**	36.8	12.0	4.4	3.8	11.6	37.6	0.4	2.1755102040816300	[0.99, 0.2, 0.6]
**40**	29.4	22.8	10.2	11.0	15.8	22.6	13.4	2.555102040816330	[0.5, 0.1, 0.7]
**0**	16.8	33.0	100.0	0.6	1.6	0.6	3.4	3.183673469387760	[0.99, 0.1, 0.1]
**39**	53.2	31.6	15.6	20.2	25.8	47.2	23.6	4.4326530612244900	[0.4, 0.1, 0.7]
**38**	84.8	45.8	23.2	37.4	46.4	82.2	62.6	7.8040816326530600	[0.3, 0.1, 0.7]
**7**	100.0	100.0	100.0	100.0	99.8	99.8	0.2	12.240816326530600	[0.99, 0.1, 0.8]
**19**	100.0	100.0	100.0	100.0	100.0	100.0	0.4	12.253061224489800	[0.99, 0.3, 0.5]
**14**	100.0	100.0	100.0	100.0	100.0	100.0	0.4	12.253061224489800	[0.99, 0.2, 0.7]
**18**	100.0	100.0	100.0	100.0	100.0	100.0	0.4	12.253061224489800	[0.99, 0.3, 0.4]
**20**	100.0	100.0	100.0	100.0	100.0	100.0	0.4	12.253061224489800	[0.99, 0.3, 0.6]
**35**	100.0	100.0	100.0	100.0	100.0	100.0	0.6	12.257142857142900	[0.99, 0.8, 0.1]
**34**	100.0	100.0	100.0	100.0	100.0	100.0	0.6	12.257142857142900	[0.99, 0.7, 0.2]
**32**	100.0	100.0	100.0	100.0	100.0	100.0	0.6	12.257142857142900	[0.99, 0.6, 0.3]
**33**	100.0	100.0	100.0	100.0	100.0	100.0	0.6	12.257142857142900	[0.99, 0.7, 0.1]
**30**	100.0	100.0	100.0	100.0	100.0	100.0	0.6	12.257142857142900	[0.99, 0.6, 0.1]
**29**	100.0	100.0	100.0	100.0	100.0	100.0	0.6	12.257142857142900	[0.99, 0.5, 0.4]
**28**	100.0	100.0	100.0	100.0	100.0	100.0	0.6	12.257142857142900	[0.99, 0.5, 0.3]
**27**	100.0	100.0	100.0	100.0	100.0	100.0	0.6	12.257142857142900	[0.99, 0.5, 0.2]
**26**	100.0	100.0	100.0	100.0	100.0	100.0	0.6	12.257142857142900	[0.99, 0.5, 0.1]
**25**	100.0	100.0	100.0	100.0	100.0	100.0	0.6	12.257142857142900	[0.99, 0.4, 0.5]
**24**	100.0	100.0	100.0	100.0	100.0	100.0	0.6	12.257142857142900	[0.99, 0.4, 0.4]
**23**	100.0	100.0	100.0	100.0	100.0	100.0	0.6	12.257142857142900	[0.99, 0.4, 0.3]
**31**	100.0	100.0	100.0	100.0	100.0	100.0	0.6	12.257142857142900	[0.99, 0.6, 0.2]
**22**	100.0	100.0	100.0	100.0	100.0	100.0	0.6	12.257142857142900	[0.99, 0.4, 0.2]
**37**	100.0	100.0	59.0	86.4	100.0	100.0	100.0	13.171428571428600	[0.2, 0.1, 0.7]
**36**	100.0	100.0	100.0	100.0	100.0	100.0	100.0	14.285714285714300	[0.1, 0.1, 0.7]

Table3-HealthyIPF_overrep_results_top_k_DE_threshold_0.4sim_215genes

	Gene_set	Term	Overlap	P-value	Adjusted P-value	Old P-value	Old Adjusted P-value	Odds Ratio	Combined Score	Genes	-log10 Adjusted P-value	-log10 FDR q-val
0	MSigDB_Hallmark_2020	Epithelial Mesenchymal Transition	16/200	5.38987249515315E-10	2.20984772301279E-08	0	0	8.564998907581390	182.7884612938860	NNMT;CXCL8;NTM;ITGA2;LAMA3;FN1;TNC;PLOD2;LAMC2;BMP1;COL4A2;COL4A1;CALD1;ABI3BP;TIMP3;MYL9	7.655637651805290	7.655637651805290
41	KEGG_2021_Human	AGE-RAGE signaling pathway in diabetic complications	10/100	1.24603509165475E-07	2.39238737597711E-05	0	0	10.67479674796750	169.7092964689730	CXCL8;COL4A2;COL4A1;AKT3;FN1;PLCE1;CCL2;NFATC1;AGER;FOXO1	4.621168497915990	4.621168497915990
42	KEGG_2021_Human	ECM-receptor interaction	8/88	4.81276187154574E-06	0.00046202513966839100	0	0	9.519323671497590	116.55687838793200	COL4A2;COL4A1;ITGA2;LAMA3;TNC;FN1;ITGB8;LAMC2	3.3353343930105000	3.3353343930105000
43	KEGG_2021_Human	Focal adhesion	11/201	1.18330915975559E-05	0.0006930856637096280	0	0	5.561016511867910	63.087566749057900	COL4A2;COL4A1;CAV1;ITGA2;AKT3;LAMA3;FN1;TNC;ITGB8;LAMC2;MYL9	3.1592130843244400	3.1592130843244400
44	KEGG_2021_Human	Amoebiasis	8/102	1.44392846606172E-05	0.0006930856637096280	0	0	8.095796073594410	90.2321644067354	CXCL8;COL4A2;IL1R1;COL4A1;LAMA3;FN1;LAMC2;RAB7A	3.1592130843244400	3.1592130843244400
1	MSigDB_Hallmark_2020	Inflammatory Response	10/200	6.3694177670215E-05	0.0013057306422394	0	0	5.030808729139920	48.604743001418400	ABCA1;HRH1;CXCL8;GCH1;IL1R1;CCL20;SLC11A2;ITGB8;CCL2;EREG	2.8841464039599900	2.8841464039599900
45	KEGG_2021_Human	Small cell lung cancer	7/92	6.08051116311413E-05	0.0023349162866358	0	0	7.799773755656110	75.71892991408990	COL4A2;COL4A1;ITGA2;AKT3;LAMA3;FN1;LAMC2	2.631728685506380	2.631728685506380
2	MSigDB_Hallmark_2020	TNF-alpha Signaling via NF-kB	9/200	0.0003246771555382070	0.0044372544590221	0	0	4.4819295481116300	36.00190242248070	ABCA1;TNFAIP8;GCH1;CCL20;DDX58;TNC;CCL2;SLC2A3;PLPP3	2.35288566549565	2.35288566549565
3	MSigDB_Hallmark_2020	Androgen Response	6/100	0.0007408899403909270	0.0075941218890069	0	0	6.013743255624550	43.3450075259520	TNFAIP8;HMGCS1;BMPR1B;NDRG1;PLPP1;FKBP5	2.119522436522230	2.119522436522230
4	MSigDB_Hallmark_2020	IL-2/STAT5 Signaling	7/199	0.0058459508177718	0.0273524867330887	0	0	3.434269831730770	17.659036167263600	SOCS2;MYO1E;P4HA1;SLC2A3;NDRG1;AGER;PLPP1	1.5630031839424600	1.5630031839424600
5	MSigDB_Hallmark_2020	Hypoxia	7/200	0.0060042044048243	0.0273524867330887	0	0	3.416301315265050	17.475390133156900	P4HA1;CAV1;S100A4;SLC2A3;ANGPTL4;NDRG1;MT1E	1.5630031839424600	1.5630031839424600
6	MSigDB_Hallmark_2020	Apical Junction	7/200	0.0060042044048243	0.0273524867330887	0	0	3.416301315265050	17.475390133156900	BMP1;ITGA2;AKT3;LAMA3;CLDN18;LAMC2;MYL9	1.5630031839424600	1.5630031839424600
7	MSigDB_Hallmark_2020	p53 Pathway	7/200	0.0060042044048243	0.0273524867330887	0	0	3.416301315265050	17.475390133156900	KRT17;SFN;S100A4;FGF13;PPM1D;NDRG1;PITPNC1	1.5630031839424600	1.5630031839424600
8	MSigDB_Hallmark_2020	KRAS Signaling Up	7/200	0.0060042044048243	0.0273524867330887	0	0	3.416301315265050	17.475390133156900	PIGR;SLPI;CCL20;ITGA2;ANGPTL4;HDAC9;EREG	1.5630031839424600	1.5630031839424600
46	KEGG_2021_Human	Human papillomavirus infection	11/331	0.0009507368332647380	0.0304235786644716	0	0	3.2799479166666700	22.822773882865200	COL4A2;COL4A1;ITGA2;AKT3;LAMA3;FN1;TNC;ITGB8;ISG15;LAMC2;FOXO1	1.516789702090360	1.516789702090360
47	KEGG_2021_Human	PI3K-Akt signaling pathway	11/354	0.0016259318303841	0.0445969873476792	0	0	3.056393986165900	19.627166386033500	PRKAA2;COL4A2;COL4A1;ITGA2;AKT3;LAMA3;FN1;TNC;ITGB8;LAMC2;EREG	1.350694478114540	1.350694478114540

Table4-SPT_overrep_results_top_k_DE_threshold_0.4sim_150genes

	Gene_set	Term	Overlap	P-value	Adjusted P-value	Old P-value	Old Adjusted P-value	Odds Ratio	Combined Score	Genes	-log10 Adjusted P-value	-log10 FDR q-val
37	KEGG_2021_Human	Herpes simplex virus 1 infection	18/498	3.83963973203034E-08	4.5307748837958E-06	0	0	5.502840909090910	93.96267148971250	ZNF320;ZNF540;ZNF221;ZNF324B;SP100;ZNF460;ZNF93;ZNF439;ZNF813;ZNF713;ZNF404;ZNF667;ZNF853;ZNF841;ZNF114;ZNF730;ZNF850;ZNF442	5.343827515652210	5.343827515652210
0	MSigDB_Hallmark_2020	TNF-alpha Signaling via NF-kB	10/200	2.77449437462695E-06	0.00010265629186119700	0	0	7.390977443609020	94.56786706856570	KLF10;NR4A1;JUN;CEBPD;MAFF;REL;MXD1;KLF4;JUNB;ATF3	3.988614427327410	3.988614427327410
38	KEGG_2021_Human	Th17 cell differentiation	6/107	0.00015608070368267900	0.009208761517278	0	0	8.147277227722770	71.4120039554682	JUN;TBX21;RORC;NFATC2;HIF1A;RUNX1	2.035798773970270	2.035798773970270
39	KEGG_2021_Human	Transcriptional misregulation in cancer	7/192	0.0006242605161553200	0.0245542469687759	0	0	5.203364203364200	38.39532673512340	HOXA10;MEIS1;MEF2C;SPI1;REL;HMGA2;RUNX1	1.6098733802992300	1.6098733802992300
40	KEGG_2021_Human	Human T-cell leukemia virus 1 infection	7/219	0.001345082255093	0.0283783020472358	0	0	4.534437260852360	29.97852556869510	JUN;SPI1;MSX2;CREB3L1;E2F2;NFATC2;CREB5	1.547013593148320	1.547013593148320
41	KEGG_2021_Human	Hepatitis B	6/162	0.0014104652862642	0.0283783020472358	0	0	5.260149572649570	34.52675723021020	JUN;CREB3L1;STAT4;E2F2;NFATC2;CREB5	1.547013593148320	1.547013593148320
42	KEGG_2021_Human	Inflammatory bowel disease	4/65	0.0014429645108763	0.0283783020472358	0	0	8.887940714125310	58.13651432264880	JUN;TBX21;RORC;STAT4	1.547013593148320	1.547013593148320

Table5-SPT_overrep_results_pluripotent

	Gene_set	Term	Overlap	P-value	Adjusted P-value	Old P-value	Old Adjusted P-value	Odds Ratio	Combined Score	Genes	-log10 Adjusted P-value	-log10 FDR q-val
14	KEGG_2021_Human	Signaling pathways regulating pluripotency of stem cells	6/143	4.83149927556573E-06	4.83149927556573E-05	0	0	15.768015925680200	193.00609254187700	DLX5;HAND1;NANOG;ISL1;POU5F1;TBX3	4.315918081242670	4.315918081242670

Table6-SPT_DNonwards_overrep_results_top_k_DE_threshold_0.4sim_64genes

	Gene_set	Term	Overlap	P-value	Adjusted P-value	Old P-value	Old Adjusted P-value	Odds Ratio	Combined Score	Genes	-log10 Adjusted P-value	-log10 FDR q-val
0	MSigDB_Hallmark_2020	TNF-alpha Signaling via NF-kB	16/200	1.74606272752118E-18	5.23818818256355E-17	0	0	35.78260869565220	1463.1211088369800	JUN;EGR2;TSC22D1;CEBPD;FOS;KLF4;RELA;KLF2;NR4A2;NR4A1;NR4A3;MAFF;FOSB;MXD1;JUNB;ATF3	16.280818903547100	16.280818903547100
1	MSigDB_Hallmark_2020	p53 Pathway	6/200	4.29336260607804E-05	0.0006440043909117060	0	0	10.527195165303900	105.85995040023400	JUN;TSC22D1;FOS;KLF4;MXD1;ATF3	3.1911111715497800	3.1911111715497800
2	MSigDB_Hallmark_2020	UV Response Up	5/158	0.00015120916641581000	0.001512091664158	0	0	10.95768250803150	96.39305070783470	NR4A1;FOSB;FOS;JUNB;ATF3	2.8204218807717500	2.8204218807717500
3	MSigDB_Hallmark_2020	Hypoxia	5/200	0.00044942710058951800	0.0026965626035371	0	0	8.579313342025210	66.1253741268086	JUN;MAFF;FOS;ETS1;ATF3	2.569189492655590	2.569189492655590
4	MSigDB_Hallmark_2020	G2-M Checkpoint	5/200	0.00044942710058951800	0.0026965626035371	0	0	8.579313342025210	66.1253741268086	TFDP1;E2F1;CTCF;E2F4;CENPA	2.569189492655590	2.569189492655590
31	KEGG_2021_Human	Human T-cell leukemia virus 1 infection	6/219	7.10667337227349E-05	0.0040478334983269	0	0	9.578921806702290	91.49681901414480	JUN;EGR2;E2F1;FOS;ETS1;RELA	2.392777359865150	2.392777359865150
30	KEGG_2021_Human	Osteoclast differentiation	5/127	5.38836573624744E-05	0.0040478334983269	0	0	13.763545429285900	135.27752950803000	JUN;FOSB;FOS;JUNB;RELA	2.392777359865150	2.392777359865150
32	KEGG_2021_Human	Pertussis	4/76	0.00010291102114390500	0.0040478334983269	0	0	18.392592592592600	168.87427081354500	JUN;IRF8;FOS;RELA	2.392777359865150	2.392777359865150
34	KEGG_2021_Human	Th1 and Th2 cell differentiation	4/92	0.00021571390025890900	0.0046079879081897	0	0	15.036363636363600	126.930329190975	JUN;TBX21;FOS;RELA	2.336488669214480	2.336488669214480
35	KEGG_2021_Human	IL-17 signaling pathway	4/94	0.00023430446990795400	0.0046079879081897	0	0	14.70074074074070	122.88186205305900	JUN;FOSB;FOS;RELA	2.336488669214480	2.336488669214480
33	KEGG_2021_Human	Hepatitis B	5/162	0.00016994315471109500	0.0046079879081897	0	0	10.676346755910600	92.67118694145990	JUN;EGR2;E2F1;FOS;RELA	2.336488669214480	2.336488669214480
36	KEGG_2021_Human	Kaposi sarcoma-associated herpesvirus infection	5/193	0.0003818861765398610	0.0056696769544984	0	0	8.901911287414350	70.0614954257495	JUN;TCF7;E2F1;FOS;RELA	2.246441685529590	2.246441685529590
37	KEGG_2021_Human	Th17 cell differentiation	4/107	0.0003843848782710840	0.0056696769544984	0	0	12.836893203883500	100.94761083790700	JUN;TBX21;FOS;RELA	2.246441685529590	2.246441685529590
38	KEGG_2021_Human	TNF signaling pathway	4/112	0.0004571198466513520	0.0059933491005399	0	0	12.239506172839500	94.12871723871910	JUN;FOS;JUNB;RELA	2.222330424786000	2.222330424786000
39	KEGG_2021_Human	Cocaine addiction	3/49	0.0005183450664787610	0.0061164717844493	0	0	21.26514611546690	160.8680528373250	JUN;FOSB;RELA	2.213499023344620	2.213499023344620
40	KEGG_2021_Human	Chemical carcinogenesis	5/239	0.0010038936429414	0.010105536532666	0	0	7.135303491235690	49.2612019818858	JUN;E2F1;FOS;KLF4;RELA	1.9954406235647700	1.9954406235647700
41	KEGG_2021_Human	Fluid shear stress and atherosclerosis	4/139	0.001027681681288	0.010105536532666	0	0	9.778271604938270	67.27890699119540	JUN;FOS;RELA;KLF2	1.9954406235647700	1.9954406235647700
44	KEGG_2021_Human	Mitophagy	3/68	0.0013503136875026	0.010387588859017	0	0	15.034804539722600	99.34124344066630	JUN;E2F1;RELA	1.9834852480871900	1.9834852480871900
45	KEGG_2021_Human	Amphetamine addiction	3/69	0.0014084866249514	0.010387588859017	0	0	14.806259314456000	97.2066380046475	JUN;FOSB;FOS	1.9834852480871900	1.9834852480871900
43	KEGG_2021_Human	Breast cancer	4/147	0.0012642410854189	0.010387588859017	0	0	9.227505827505830	61.57776025614860	JUN;TCF7;E2F1;FOS	1.9834852480871900	1.9834852480871900
42	KEGG_2021_Human	Inflammatory bowel disease	3/65	0.0011849578216224	0.010387588859017	0	0	15.76467477525120	106.22313689436600	JUN;TBX21;RELA	1.9834852480871900	1.9834852480871900
46	KEGG_2021_Human	Cellular senescence	4/156	0.0015732312737645	0.010920075900248	0	0	8.677192982456140	56.008014940620400	E2F1;E2F4;ETS1;RELA	1.9617743430466400	1.9617743430466400
47	KEGG_2021_Human	Leishmaniasis	3/77	0.0019310474335392	0.0126590887309794	0	0	13.200265839610100	82.49760520901610	JUN;FOS;RELA	1.897597556036770	1.897597556036770
48	KEGG_2021_Human	B cell receptor signaling pathway	3/81	0.0022319579660503	0.0138616336838915	0	0	12.520807061790700	76.43797537174070	JUN;FOS;RELA	1.8581855822719700	1.8581855822719700
49	KEGG_2021_Human	Colorectal cancer	3/86	0.002647000027125	0.0156173001600379	0	0	11.763578905787100	69.80893974501690	JUN;TCF7;FOS	1.8063940426060900	1.8063940426060900
50	KEGG_2021_Human	PD-L1 expression and PD-1 checkpoint pathway in cancer	3/89	0.0029173912116181	0.0163929601414733	0	0	11.3515059092642	66.25948331984450	JUN;FOS;RELA	1.7853426170805100	1.7853426170805100
6	MSigDB_Hallmark_2020	KRAS Signaling Up	4/200	0.0038612882755342	0.0165483783237183	0	0	6.714285714285710	37.30963669275870	ZNF639;IRF8;KLF4;ETS1	1.78124455896186	1.78124455896186
5	MSigDB_Hallmark_2020	IL-2/STAT5 Signaling	4/199	0.0037930508280137	0.0165483783237183	0	0	6.749059829059830	37.6232050947777	EOMES;MAFF;IRF8;MXD1	1.78124455896186	1.78124455896186
51	KEGG_2021_Human	Prostate cancer	3/97	0.0037195964427495	0.0199505627383838	0	0	10.381234740146500	58.07408154439390	TCF7;E2F1;RELA	1.7000448498154300	1.7000448498154300
52	KEGG_2021_Human	Chagas disease	3/102	0.0042828592511413	0.0205222846166626	0	0	9.85444610034774	53.73761944749820	JUN;FOS;RELA	1.687774293600170	1.687774293600170
53	KEGG_2021_Human	T cell receptor signaling pathway	3/104	0.0045218593223155	0.0205222846166626	0	0	9.65833468592761	52.14372651615840	JUN;FOS;RELA	1.687774293600170	1.687774293600170
55	KEGG_2021_Human	C-type lectin receptor signaling pathway	3/104	0.0045218593223155	0.0205222846166626	0	0	9.65833468592761	52.14372651615840	JUN;EGR2;RELA	1.687774293600170	1.687774293600170
54	KEGG_2021_Human	Toll-like receptor signaling pathway	3/104	0.0045218593223155	0.0205222846166626	0	0	9.65833468592761	52.14372651615840	JUN;FOS;RELA	1.687774293600170	1.687774293600170
56	KEGG_2021_Human	Herpes simplex virus 1 infection	6/498	0.0051187971017643	0.0223710391854887	0	0	4.088309503784690	21.565161367184100	ZNF726;ZNF736;ZNF433;RELA;ZNF442;ZNF563	1.6503138414715000	1.6503138414715000
57	KEGG_2021_Human	Pathways in cancer	6/531	0.0069472755724466	0.0292778041981679	0	0	3.824827586206900	19.007120009269000	JUN;TCF7;E2F1;FOS;ETS1;RELA	1.5334614979817600	1.5334614979817600
58	KEGG_2021_Human	Cell cycle	3/124	0.0073603848624526	0.0299491521989452	0	0	8.053786749762910	39.55732576812040	TFDP1;E2F1;E2F4	1.5236154671154500	1.5236154671154500
59	KEGG_2021_Human	Relaxin signaling pathway	3/129	0.0082019467300635	0.03163210104052	0	0	7.73224043715847	37.14091804206520	JUN;FOS;RELA	1.4998719607874100	1.4998719607874100
60	KEGG_2021_Human	Salmonella infection	4/249	0.0083101282394586	0.03163210104052	0	0	5.358095238095240	25.66677773392000	JUN;TCF7;FOS;RELA	1.4998719607874100	1.4998719607874100
61	KEGG_2021_Human	Yersinia infection	3/137	0.0096621269756263	0.0356290932226222	0	0	7.267678003425500	33.718693496092400	JUN;FOS;RELA	1.4481952305095700	1.4481952305095700
62	KEGG_2021_Human	Measles	3/139	0.0100493205129382	0.0359339339553549	0	0	7.160077145612340	32.93814673221920	JUN;FOS;RELA	1.4444952347414000	1.4444952347414000
63	KEGG_2021_Human	Apoptosis	3/142	0.0106468909590826	0.0369509745050515	0	0	7.004481660573180	31.81776939369300	JUN;FOS;RELA	1.4323741035050000	1.4323741035050000
64	KEGG_2021_Human	Cushing syndrome	3/155	0.0134718225129174	0.044157640459007	0	0	6.401207937877480	27.57099475060910	NR4A1;TCF7;E2F1	1.3549941409629800	1.3549941409629800
65	KEGG_2021_Human	Non-alcoholic fatty liver disease	3/155	0.0134718225129174	0.044157640459007	0	0	6.401207937877480	27.57099475060910	JUN;FOS;RELA	1.3549941409629800	1.3549941409629800
66	KEGG_2021_Human	MAPK signaling pathway	4/294	0.0145782354931802	0.0464927510323046	0	0	4.51632183908046	19.09602753718230	NR4A1;JUN;FOS;RELA	1.3326147553268900	1.3326147553268900

Table7-CD8_DNonwards_overrep_results_top_k_DE_threshold_0.4sim_61genes

	Gene_set	Term	Overlap	P-value	Adjusted P-value	Old P-value	Old Adjusted P-value	Odds Ratio	Combined Score	Genes	-log10 Adjusted P-value	-log10 FDR q-val
0	MSigDB_Hallmark_2020	TNF-alpha Signaling via NF-kB	14/200	9.41556644203802E-16	2.63635860377065E-14	0	0	31.63372226035230	1094.495066767000	JUN;TSC22D1;CEBPD;FOS;KLF4;KLF2;NR4A2;NR4A1;NR4A3;FOSB;HES1;MXD1;JUNB;ATF3	13.5789951629110	13.5789951629110
1	MSigDB_Hallmark_2020	p53 Pathway	7/200	2.48680877848643E-06	3.481532289881E-05	0	0	13.26252158894650	171.14634624328500	JUN;TSC22D1;ZBTB16;FOS;KLF4;MXD1;ATF3	4.458229572554430	4.458229572554430
2	MSigDB_Hallmark_2020	UV Response Up	5/158	0.00012024964289799600	0.0011223300003812	0	0	11.54645191409900	104.21758933270900	NR4A1;FOSB;FOS;JUNB;ATF3	2.949879428041880	2.949879428041880
3	MSigDB_Hallmark_2020	KRAS Signaling Up	5/200	0.0003592601567844900	0.0025148210974914	0	0	9.04029304029304	71.70275663984070	MAFB;PRDM1;KLF4;ETS1;ETV5	2.5994929048966600	2.5994929048966600
4	MSigDB_Hallmark_2020	Hypoxia	4/200	0.0032458250655199	0.0129833002620798	0	0	7.0687432867884	40.50662547348920	JUN;FOS;ETS1;ATF3	1.886614898950970	1.886614898950970
5	MSigDB_Hallmark_2020	Estrogen Response Early	4/200	0.0032458250655199	0.0129833002620798	0	0	7.0687432867884	40.50662547348920	HES1;FOS;KLF4;MYBL1	1.886614898950970	1.886614898950970
6	MSigDB_Hallmark_2020	E2F Targets	4/200	0.0032458250655199	0.0129833002620798	0	0	7.0687432867884	40.50662547348920	HMGA1;PA2G4;CTCF;MXD3	1.886614898950970	1.886614898950970
28	KEGG_2021_Human	Th1 and Th2 cell differentiation	4/92	0.00017904509648552900	0.0152188332012699	0	0	15.830143540669900	136.5804656373480	JUN;TBX21;FOS;RUNX3	1.8176186428134500	1.8176186428134500
29	KEGG_2021_Human	Osteoclast differentiation	4/127	0.0006116673121719760	0.0259958607673089	0	0	11.305662530309500	83.65423783356070	JUN;FOSB;FOS;JUNB	1.585095797760950	1.585095797760950
30	KEGG_2021_Human	Breast cancer	4/147	0.0010561688730801	0.026045739380103	0	0	9.71463624095203	66.57544345938670	JUN;HEY1;HES1;FOS	1.5842633094103600	1.5842633094103600
31	KEGG_2021_Human	Amphetamine addiction	3/69	0.0012256818531813	0.026045739380103	0	0	15.574451410658300	104.41514007609600	JUN;FOSB;FOS	1.5842633094103600	1.5842633094103600
7	MSigDB_Hallmark_2020	Reactive Oxygen Species Pathway	2/49	0.0098141695602493	0.0343495934608726	0	0	14.346916696718400	66.33911074696530	HHEX;JUNB	1.464078398596990	1.464078398596990
32	KEGG_2021_Human	Transcriptional misregulation in cancer	4/192	0.0028035057218447	0.0360764762121325	0	0	7.372527062336690	43.32749076633030	HHEX;NR4A3;ZBTB16;ETV5	1.4427758889893900	1.4427758889893900
33	KEGG_2021_Human	Maturity onset diabetes of the young	2/26	0.0028369216422226	0.0360764762121325	0	0	28.12853107344630	164.97484015733700	HHEX;HES1	1.4427758889893900	1.4427758889893900
34	KEGG_2021_Human	IL-17 signaling pathway	3/94	0.002971003923352	0.0360764762121325	0	0	11.28154604016670	65.64568466694600	JUN;FOSB;FOS	1.4427758889893900	1.4427758889893900
35	KEGG_2021_Human	Herpes simplex virus 1 infection	6/498	0.0040345587141162	0.0363613285588977	0	0	4.311973392461200	23.771298512001500	ZNF726;ZNF433;ZNF257;ZNF135;ZNF442;ZFP28	1.4393602570436600	1.4393602570436600
36	KEGG_2021_Human	Parathyroid hormone synthesis, secretion and action	3/106	0.0041668256176041	0.0363613285588977	0	0	9.96116504854369	54.59316889336490	NR4A2;MAFB;FOS	1.4393602570436600	1.4393602570436600
37	KEGG_2021_Human	Th17 cell differentiation	3/107	0.0042778033598703	0.0363613285588977	0	0	9.864887267904510	53.80620886100000	JUN;TBX21;FOS	1.4393602570436600	1.4393602570436600
38	KEGG_2021_Human	TNF signaling pathway	3/112	0.0048596216360786	0.0375516217333352	0	0	9.409996836444160	50.12512124489080	JUN;FOS;JUNB	1.4253713024779200	1.4253713024779200
39	KEGG_2021_Human	Pathways in cancer	6/531	0.0054980945115165	0.0389448361232423	0	0	4.034077922077920	20.990734278801100	JUN;HEY1;ZBTB16;HES1;FOS;ETS1	1.4095501192970800	1.4095501192970800
40	KEGG_2021_Human	Chemical carcinogenesis	4/239	0.0060923209366526	0.039834406124267	0	0	5.883986562150060	30.01260420430120	JUN;KLF5;FOS;KLF4	1.3997416531783800	1.3997416531783800

Table8-ATO_manifest

TO_number	Fiaj	Kolf	MS5hDLL4	iPSC source	human_cell_batch	ATO_day	ATO_wk	treatment	Date_of_sample	FACS_sort	10x_kit	sanger_sample_ID	sequence_name	CITE_name
1	Y	Y	Y	CGaP	souporcell_Fiaj_Kolf	-14	-2	None	28/01/2020	unsorted_sorted	5' v1.0	T01_D-14	6180STDY8814872	6180STDY8814874
2	Y	N	Y	CGaP	souporcell_Fiaj_Kolf	-7	-1	None	04/02/2020	unsorted	5' v1.0	T01_D-7	6180STDY8814873	
3	Y	Y	N	CGaP	souporcell_Fiaj_Kolf	7	1	None	26/08/2020	mouseCD29neg	5' v1.0	TO3 KOLF_FIAJ D7	6180STDY9448806	6180STDY9448807
4	Y	Y	N	CGaP	souporcell_Fiaj_Kolf	20	3	None	08/09/2020	mouseCD29neg_CD45pos	5' v1.0	TO4 KOLF_FIAJ wk3	6180STDY9448808	6180STDY9448810
5	Y	Y	Y	CGaP	souporcell_Fiaj_Kolf	37	5	None	25/09/2020	live	5' v1.0	T05 KOLF_FIAJ wk5	6180STDY9448811	
6	Y	Y	Y	CGaP	souporcell_Fiaj_Kolf	50	7	None	08/10/2020	live	5' v1.0	T06 KOLF_FIAJ wk7	6180STDY9448813	6180STDY9448815
9	Y	Y	N	CGaP	souporcell_Fiaj_Kolf	-7	-1	None	26/04/2021	mouse CD29neg	5' v1.0	T09_Kolf_Fiaj_ATO_WT_D-7	6180STDY10267835	
10	Y	Y	N	CGaP	souporcell_Fiaj_Kolf	0	0	None	04/05/2021	mouse CD29neg	5' v1.0	T10_Kolf_Fiaj_ATO_WT_D0	6180STDY10267836	
23	N	Y	Y	CGaP	Kolf	49	7	TNFa	15/02/2023	CD45pos	5' v2.0	T23_TNF_wk7	6180STDY13657795	6180STDY13657796

Table9-ATO_hashtagging

TO_number	iPSC line	iPSC source	iPSC passage	ATO days	treatment	Date_of_sample	FACS_sort	Hashtag antibody	sanger_sample_ID	sequence_name	CITE_name
1	Fiaj_1	CGaP	P21	-14	None	28/01/2020	unsorted	TotalSeq-C0251	T01 D-14	6180STDY8814872	6180STDY8814874
1	Kolf_2	CGaP	P21	-14	None	28/01/2020	unsorted	TotalSeq-C0252	T01 D-14	6180STDY8814872	6180STDY8814874
1	Kolf_2	CGaP	P21	-14	None	28/01/2020	CD326-CD56+	TotalSeq-C0253	T01 D-14	6180STDY8814872	6180STDY8814874
1	Fiaj_1	CGaP	P21	-14	None	28/01/2020	total - CD326-CD56+	TotalSeq-C0254	T01 D-14	6180STDY8814872	6180STDY8814874
1	Kolf_2	CGaP	P21	-14	None	28/01/2020	total - CD326-CD56+	TotalSeq-C0255	T01 D-14	6180STDY8814872	6180STDY8814874
1	MS5-hDLL4	Gay Crooks	PN+4	NA	None		NA	NA	T01 D-14	6180STDY8814872	6180STDY8814874
3	Kolf_2	CGaP	P23	6	None	26/08/2020	mouse CD29-	TotalSeq-C0253	TO3 KOLF_FIAJ D7	6180STDY9448806	6180STDY9448807
3	Fiaj_1	CGaP	P23	7	None	26/08/2020	mouse CD29-	TotalSeq-C0251	TO3 KOLF_FIAJ D7	6180STDY9448806	6180STDY9448807
23	Kolf_2	CGaP		49	None	15/02/2023	CD45+	TotalSeq-C0251	T23_TNF_wk7	6180STDY13657795	6180STDY13657796
23	Kolf_2	CGaP		49	TNF1ng/ml	15/02/2023	CD45+	TotalSeq-C0252	T23_TNF_wk7	6180STDY13657795	6180STDY13657796
23	Kolf_2	CGaP		49	TNF5ng/ml	15/02/2023	CD45+	TotalSeq-C0253	T23_TNF_wk7	6180STDY13657795	6180STDY13657796
23	Kolf_2	CGaP		49	TNF25ng/ml	15/02/2023	CD45+	TotalSeq-C0254	T23_TNF_wk7	6180STDY13657795	6180STDY13657796

Table10-Visium_TNFa_expression

	slide 1	slide 1	slide 1	slide 1	slide 2	slide 2	slide 2	slide 2	slide 3	slide 3	slide 3	slide 3
	mean_cortex	mean_medulla	medulla_high	ransums_pval	mean_cortex	mean_medulla	medulla_high	ransums_pval	mean_cortex	mean_medulla	medulla_high	ransums_pval
**NR4A1**	0.25	0.58	TRUE	1.54E-05	0.69	2.72	TRUE	9.31E-52	0.23	1.1	TRUE	1.53E-22
**JUNB**	0.41	0.87	TRUE	1.74E-10	0.89	2.05	TRUE	1.22E-30	0.43	1.18	TRUE	2.17E-18
**ZBTB10**	0.3	0.37	TRUE	2.75E-01	0.35	0.6	TRUE	4.42E-06	0.28	0.37	TRUE	1.37E-01
**IRF1**	0.65	1.09	TRUE	1.82E-07	1.16	3.45	TRUE	8.58E-66	0.64	1.49	TRUE	1.22E-17
**KLF6**	0.61	0.9	TRUE	5.27E-04	1.05	1.74	TRUE	3.94E-16	0.78	1.35	TRUE	1.51E-08
**NR4A2**	0.05	0.1	TRUE	3.78E-01	0.08	0.35	TRUE	9E-10	0.02	0.1	TRUE	9.05E-02
**FOS**	0.23	0.48	TRUE	2.47E-03	0.47	0.95	TRUE	7.85E-11	0.21	0.33	TRUE	2.68E-02
**FOSB**	0.03	0.03	TRUE	8.73E-01	0.03	0.03	TRUE	8.83E-01	0.01	0.01	TRUE	9.39E-01
**REL**	0.09	0.13	TRUE	3.07E-01	0.08	0.22	TRUE	3.91E-04	0.09	0.22	TRUE	5.66E-03
**FOSL2**	0.16	0.31	TRUE	1.31E-03	0.32	0.85	TRUE	8.57E-18	0.1	0.32	TRUE	2.21E-05
**EGR2**	0.07	0.11	TRUE	2.78E-01	0.09	0.32	TRUE	2.69E-06	0.05	0.15	TRUE	5.45E-02
**NR4A3**	0.06	0.16	TRUE	2.81E-02	0.11	0.54	TRUE	8.63E-16	0.05	0.33	TRUE	5.75E-07
**BHLHE40**	0.32	0.72	TRUE	2.75E-07	0.8	2.42	TRUE	1.03E-42	0.28	1.34	TRUE	3.63E-29
**NFE2L2**	0.52	0.85	TRUE	1.39E-05	0.84	1.63	TRUE	1.35E-19	0.52	1.13	TRUE	2.67E-14
**NFKB2**	0.25	0.78	TRUE	9.83E-11	0.65	2.34	TRUE	2.41E-54	0.23	1.1	TRUE	3.18E-23
**RELB**	0.21	0.43	TRUE	1.39E-03	0.37	1.26	TRUE	1.98E-33	0.22	0.91	TRUE	2.05E-18
**CD27**	0.34	1	TRUE	2.34E-16	0.69	3.93	TRUE	4.59E-97	0.32	2.28	TRUE	9.78E-36
**RAG1**	6.54	4.49	FALSE	3.4E-12	9.7	4.24	FALSE	8.43E-61	7.54	4.05	FALSE	1.15E-29
**RAG2**	0.95	0.57	FALSE	1.35E-07	1.2	0.46	FALSE	1.27E-28	1.07	0.41	FALSE	1.84E-17

Supplementary Table 10 | Expression of selected TNFα pathway and marker genes in fetal thymus Visium data.

Each column represents the results for each gene. The table includes individual results for each slide across 3 fetal thymic Visium slides from the developing human immune atlas.

[mean_cortex] The average of UMI counts for that gene in regions labelled as thymic cortex

[mean_medulla] The average of UMI counts for that gene in regions labelled as thymic medulla

[medulla_high] Is TRUE if the mean_medulla is higher than mean_cortex for that gene

[ranksums_pval] P-value from Wilcoxon rank-sum test for UMI counts for that gene in all cortex regions vs. UMI counts in all medulla regions
