# Supplementary material for: Gene-level alignment of single-cell trajectories
Source: Nat Methods. 2024 Sep 19;22(1):68–81. doi: 10.1038/s41592-024-02378-4 (PMC11725504; doi:10.1038/s41592-024-02378-4)
Supplement: Supplementary file 2 — Reporting Summary [file 41592_2024_2378_MOESM2_ESM.pdf]

nature portfolio

Corresponding author(s): Sarah A. Teichmann

Last updated by author(s): 14th May 2024

Reporting Summary

Nature Portfolio wishes to improve the reproducibility of the work that we publish. This form provides structure for consistency and transparency in reporting. For further information on Nature Portfolio policies, see our Editorial Policies and the Editorial Policy Checklist.

Statistics

For all statistical analyses, confirm that the following items are present in the figure legend, table legend, main text, or Methods section.

n/a	Confirmed	
☐	☒	The exact sample size (*n*) for each experimental group/condition, given as a discrete number and unit of measurement
☐	☒	A statement on whether measurements were taken from distinct samples or whether the same sample was measured repeatedly
☐	☒	The statistical test(s) used AND whether they are one- or two-sided<br>*Only common tests should be described solely by name; describe more complex techniques in the Methods section.*
☐	☒	A description of all covariates tested
☐	☒	A description of any assumptions or corrections, such as tests of normality and adjustment for multiple comparisons
☐	☒	A full description of the statistical parameters including central tendency (e.g. means) or other basic estimates (e.g. regression coefficient) AND variation (e.g. standard deviation) or associated estimates of uncertainty (e.g. confidence intervals)
☐	☒	For null hypothesis testing, the test statistic (e.g. *F*, *t*, *r*) with confidence intervals, effect sizes, degrees of freedom and *P* value noted<br>*Give P values as exact values whenever suitable.*
☐	☒	For Bayesian analysis, information on the choice of priors and Markov chain Monte Carlo settings
☒	☐	For hierarchical and complex designs, identification of the appropriate level for tests and full reporting of outcomes
☒	☐	Estimates of effect sizes (e.g. Cohen's *d*, Pearson's *r*), indicating how they were calculated

Our web collection on statistics for biologists contains articles on many of the points above.

Software and code

Policy information about availability of computer code

Data collection	No software was used during data collection
Data analysis	We used the following Python software libraries for our analyses and software development.<br><br>Python (v3.8), cellranger (v3.0.2), souporcell (v2.4.0), scrublet (v0.2.3), scanpy (v1.9.6), scvi (v0.14.5), pyro (v1.8.0), bbknn (v1.5.1), celltypist (v0.1.4), leven (v1.0.4), scipy (v1.10.1), optBinning (v0.18.0), gpytorch (v1.5.1), statsmodels (v0.13.5), gseapy (v1.0.4), cellalign (v0.1.0), sklearn (v1.2.2), seaborn >=v0.12.2 pandas>=v2.0.3, regex >= v2.5.135, matplotlib >= v3.7.1, numpy<v2.<br><br>Code availability: All Python source code and data analysis notebooks are publicly available at: https://github.com/Teichlab/Genes2Genes and https://github.com/Teichlab/G2G_notebooks. Genes2Genes is implemented as an open-source Python package. Our GitHub repository provides installation instructions and technical documentation for interpreting results.

For manuscripts utilizing custom algorithms or software that are central to the research but not yet described in published literature, software must be made available to editors and reviewers. We strongly encourage code deposition in a community repository (e.g. GitHub). See the Nature Portfolio guidelines for submitting code & software for further information.

Data

Policy information about availability of data

All manuscripts must include a data availability statement. This statement should provide the following information, where applicable:

- Accession codes, unique identifiers, or web links for publicly available datasets
- A description of any restrictions on data availability
- For clinical datasets or third party data, please ensure that the statement adheres to our policy

Data used to perform analyses in the manuscript are available at: https://zenodo.org/records/11182400 and https://github.com/Teichlab/G2G_notebooks. All generated alignments are available as Supplementary Data. Raw sequencing data for newly generated sequencing libraries have been deposited in ArrayExpress (accession number E-MTAB-12720).

Human research participants

Policy information about studies involving human research participants and Sex and Gender in Research.

Reporting on sex and gender	N/A
Population characteristics	N/A
Recruitment	N/A
Ethics oversight	N/A

Note that full information on the approval of the study protocol must also be provided in the manuscript.

Field-specific reporting

Please select the one below that is the best fit for your research. If you are not sure, read the appropriate sections before making your selection.

☒ Life sciences ☐ Behavioural & social sciences ☐ Ecological, evolutionary & environmental sciences

For a reference copy of the document with all sections, see nature.com/documents/nr-reporting-summary-flat.pdf

Life sciences study design

All studies must disclose on these points even when the disclosure is negative.

Sample size	No sample size calculations were performed. Sample sizes were determined based on the availability of datasets. When sub-sampling from each cell population, we defined a sufficient minimum number of 500 samples, otherwise the small populations were retained.
Data exclusions	No data were excluded.
Replication	Our artificial thymic organoid had two different iPSC lines. Downloaded Pan fetal reference (Suo et al. 2022) had 33 batches (due to multiple donors and 3' vs 5' 10X chemistry). Downloaded Healthy/IPF datasets (Adams et al. 2020) had 28 donors and 31 donors, respectively. Downloaded PAM/LPS and mouse pancreas trajectory datasets did not have the notion of replicates.
Randomization	N/A as genome-wide single cell RNA-sequencing was performed unbiased without needing any a priori information
Blinding	N/A as genome-wide single cell RNA-sequencing was performed unbiased without needing any a priori information

Reporting for specific materials, systems and methods

We require information from authors about some types of materials, experimental systems and methods used in many studies. Here, indicate whether each material, system or method listed is relevant to your study. If you are not sure if a list item applies to your research, read the appropriate section before selecting a response.

Materials & experimental systems

n/a	Involved in the study
☐	☒ Antibodies
☐	☒ Eukaryotic cell lines
☒	☐ Palaeontology and archaeology
☒	☐ Animals and other organisms
☒	☐ Clinical data
☒	☐ Dual use research of concern

Methods

n/a	Involved in the study
☒	☐ ChIP-seq
☐	☒ Flow cytometry
☒	☐ MRI-based neuroimaging

Antibodies

Antibodies used

PE anti-human CD326 antibody, Biolegend, 324205
APC anti-human CD56 antibody, Biolegend, 318309
APC/Cy7 anti-mouse CD29 antibody, Biolegend, 102225
BV785 anti-human CD45 antibody, Biolegend, 304047
TotalSeq-C0251, Biolegend, 394661
TotalSeq-C0252, Biolegend, 394663
TotalSeq-C0253, Biolegend, 394665
TotalSeq-C0254, Biolegend, 394667
TotalSeq-C0255, Biolegend, 394669

Validation

All antibodies were obtained from commercial vendors and were validated by the vendors.
PE anti-human CD326 antibody, Biolegend, 324205
https://d1spbj2x7qk4bg.cloudfront.net/en-gb/products/pe-anti-human-cd326-epcam-antibody-3757?pdf=true&displayInline=true&leftRightMargin=15&topBottomMargin=15&filename=PE%20anti-human%20CD326%20(EpCAM)%20Antibody.pdf&v=20240411093413

APC anti-human CD56 antibody, Biolegend, 318309
https://d1spbj2x7qk4bg.cloudfront.net/en-ie/products/apc-anti-human-cd56-ncam-antibody-3798?pdf=true&displayInline=true&leftRightMargin=15&topBottomMargin=15&filename=APC%20anti-human%20CD56%20(NCAM)%20Antibody.pdf&v=20240412063148

APC/Cy7 anti-mouse CD29 antibody, Biolegend, 102225
https://d1spbj2x7qk4bg.cloudfront.net/en-gb/products/apc-cyanine7-anti-mouse-rat-cd29-antibody-6184?pdf=true&displayInline=true&leftRightMargin=15&topBottomMargin=15&filename=APC/Cyanine7%20anti-mouse/rat%20CD29%20Antibody.pdf&v=20240410063626

BV785 anti-human CD45 antibody, Biolegend, 304047
https://d1spbj2x7qk4bg.cloudfront.net/nl-nl/products/brilliant-violet-785-anti-human-cd45-antibody-9325?pdf=true&displayInline=true&leftRightMargin=15&topBottomMargin=15&filename=Brilliant%20Violet%20785%E2%84%A2%20anti-human%20CD45%20Antibody.pdf&v=20240411093413

TotalSeq-C0251, Biolegend, 394661
https://d1spbj2x7qk4bg.cloudfront.net/nl-nl/products/totalseq-c0251-anti-human-hashtag-1-antibody-17162?pdf=true&displayInline=true&leftRightMargin=15&topBottomMargin=15&filename=TotalSeq%E2%84%A2-C0251%20anti-human%20Hashtag%201%20Antibody.pdf&v=20240208073156

TotalSeq-C0252, Biolegend, 394663
https://d1spbj2x7qk4bg.cloudfront.net/nl-nl/products/totalseq-c0252-anti-human-hashtag-2-antibody-17163?pdf=true&displayInline=true&leftRightMargin=15&topBottomMargin=15&filename=TotalSeq%E2%84%A2-C0252%20anti-human%20Hashtag%202%20Antibody.pdf&v=20240208073156

TotalSeq-C0253, Biolegend, 394665
https://d1spbj2x7qk4bg.cloudfront.net/nl-nl/products/totalseq-c0253-anti-human-hashtag-3-antibody-17164?pdf=true&displayInline=true&leftRightMargin=15&topBottomMargin=15&filename=TotalSeq%E2%84%A2-C0253%20anti-human%20Hashtag%203%20Antibody.pdf&v=20240208073156

TotalSeq-C0254, Biolegend, 394667
https://d1spbj2x7qk4bg.cloudfront.net/nl-nl/products/totalseq-c0254-anti-human-hashtag-4-antibody-17165?pdf=true&displayInline=true&leftRightMargin=15&topBottomMargin=15&filename=TotalSeq%E2%84%A2-C0254%20anti-human%20Hashtag%204%20Antibody.pdf&v=20240208073156

TotalSeq-C0255, Biolegend, 394669
https://d1spbj2x7qk4bg.cloudfront.net/nl-nl/products/totalseq-c0255-anti-human-hashtag-5-antibody-17166?pdf=true&displayInline=true&leftRightMargin=15&topBottomMargin=15&filename=TotalSeq%E2%84%A2-C0255%20anti-human%20Hashtag%205%20Antibody.pdf&v=20240208073156

Eukaryotic cell lines

Policy information about cell lines and Sex and Gender in Research

Cell line source(s)	MS5 line transduced with human DLL4 was obtained from G. Crooks (UCLA) as a gift. Two iPSC lines were used in this study. Cell lines HPSI0114i-kolf_2 (Kolf) and HPSI0514i-fiaj_1 (Fiaj) were obtained from the Human Induced Pluripotent Stem Cell initiative (HipSci: www.hipsci.org) collection.
Authentication	None of the cell lines used were authenticated.
Mycoplasma contamination	All lines were tested negative for mycoplasma contamination.
Commonly misidentified lines (See ICLAC register)	Nil

Flow Cytometry

Plots

Confirm that:

☒ The axis labels state the marker and fluorochrome used (e.g. CD4-FITC).

☒ The axis scales are clearly visible. Include numbers along axes only for bottom left plot of group (a 'group' is an analysis of identical markers).

☒ All plots are contour plots with outliers or pseudocolor plots.

☒ A numerical value for number of cells or percentage (with statistics) is provided.

Methodology

Sample preparation	Cells were harvested by centrifugation, resuspended in FACS buffer (PBS + 0.5% FBS + 2mM EDTA) and stained with antibody mixes for 30 min at 4 degree celsius. All antibodies used were added in as 2 µl per antibody in a total of 100 µl staining solution. Cells were then washed once with FACS buffer before flow cytometry.
Instrument	Beckman Coulter CytoFLEX, BD Influx, ThermoFisher Bigfoot Spectral Cell sorters
Software	Manufacturer's default softward was used. Analysis was done in Flowjo v10.
Cell population abundance	Shown in Supplementary Fig.7.
Gating strategy	For flow cytometry gating, cells were gated on FSC/SSC; then SSC-W vs SSC-H or FSC-W vs FSC-A for singlets; and individual stains were gated based on negative controls.

☒ Tick this box to confirm that a figure exemplifying the gating strategy is provided in the Supplementary Information.
