# Supplementary figures and images for: Gene-level alignment of single-cell trajectories
Source: Nat Methods. 2024 Sep 19;22(1):68–81. doi: 10.1038/s41592-024-02378-4 (PMC11725504; doi:10.1038/s41592-024-02378-4)

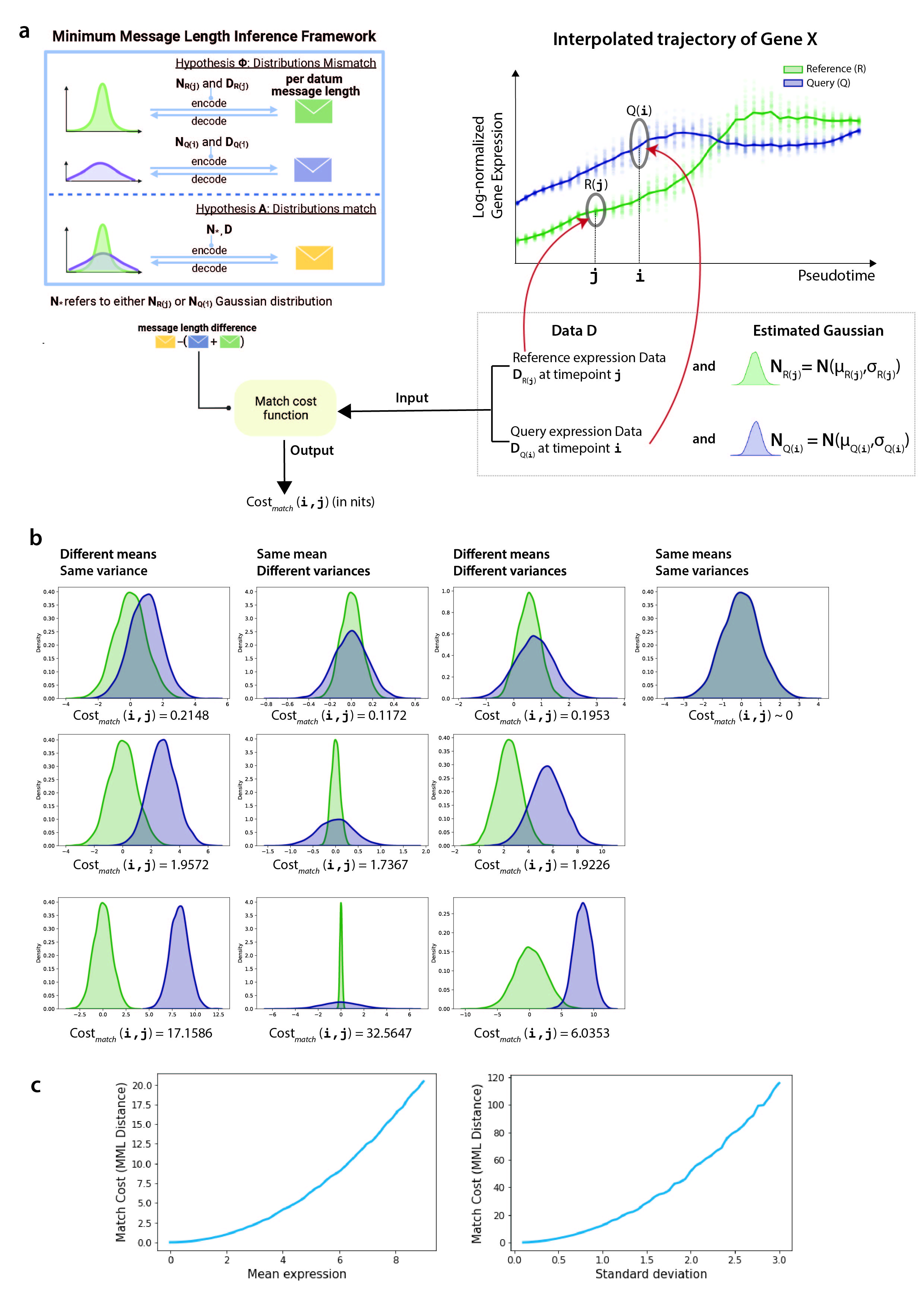

Supplement: Supplementary file 4 — G2G alignment data and Supplementary Fig. files. [file 41592_2024_2378_MOESM4_ESM.zip › SupplementaryData/SupplementaryFigures/SupplementaryFig1.jpg]

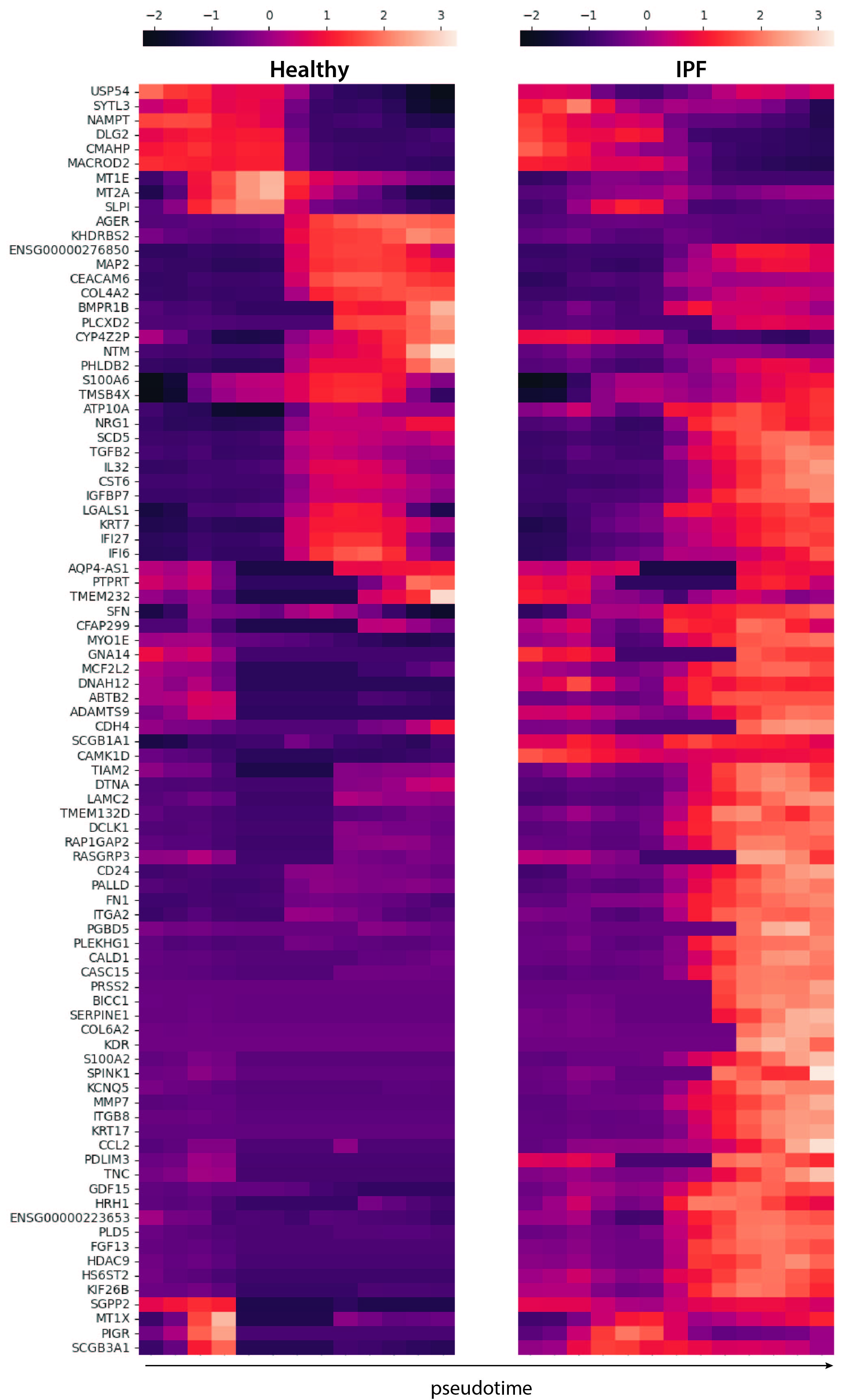

Supplement: Supplementary file 4 — G2G alignment data and Supplementary Fig. files. [file 41592_2024_2378_MOESM4_ESM.zip › SupplementaryData/SupplementaryFigures/SupplementaryFig3.jpg]

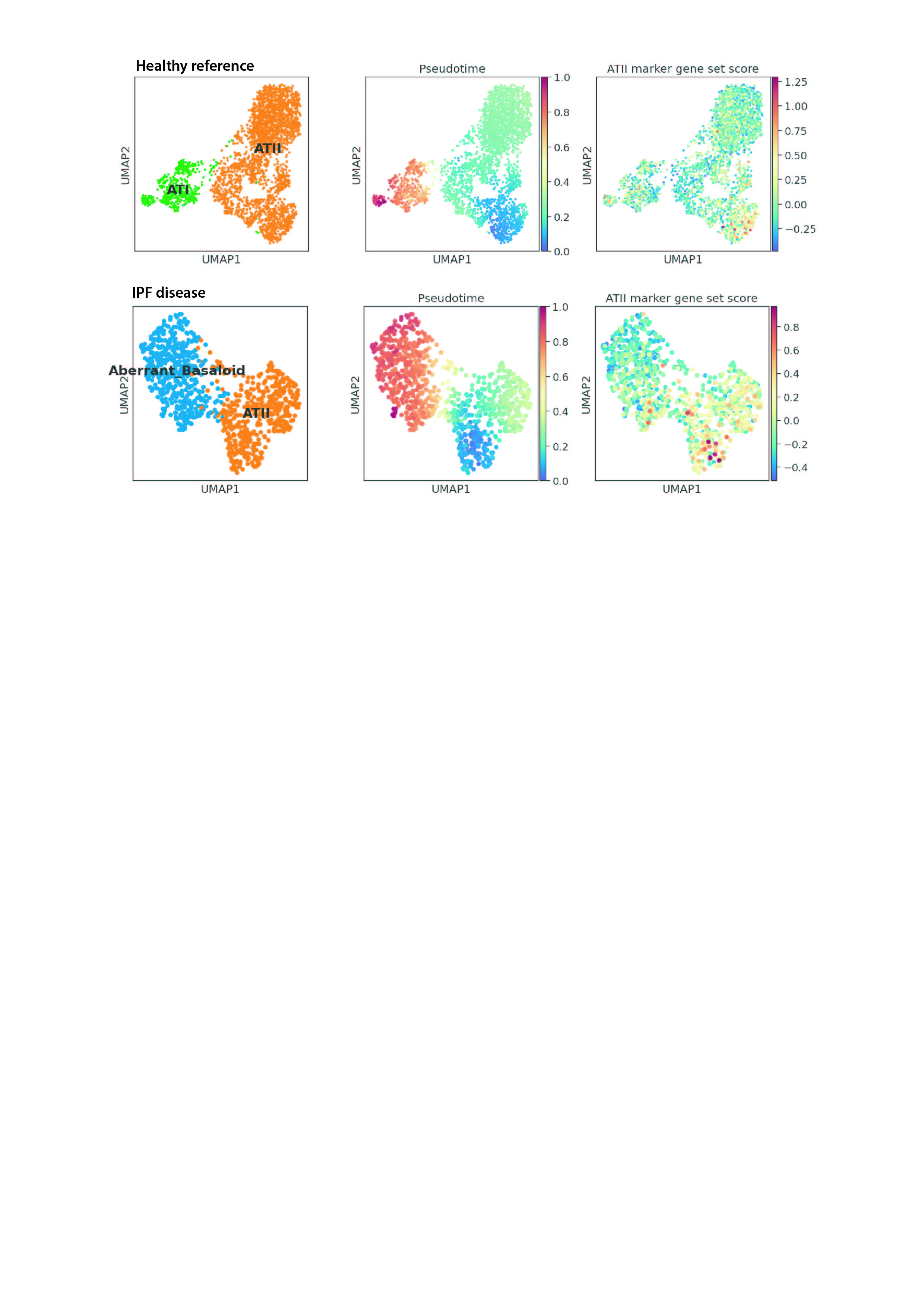

Supplement: Supplementary file 4 — G2G alignment data and Supplementary Fig. files. [file 41592_2024_2378_MOESM4_ESM.zip › SupplementaryData/SupplementaryFigures/SupplementaryFig2.jpg]

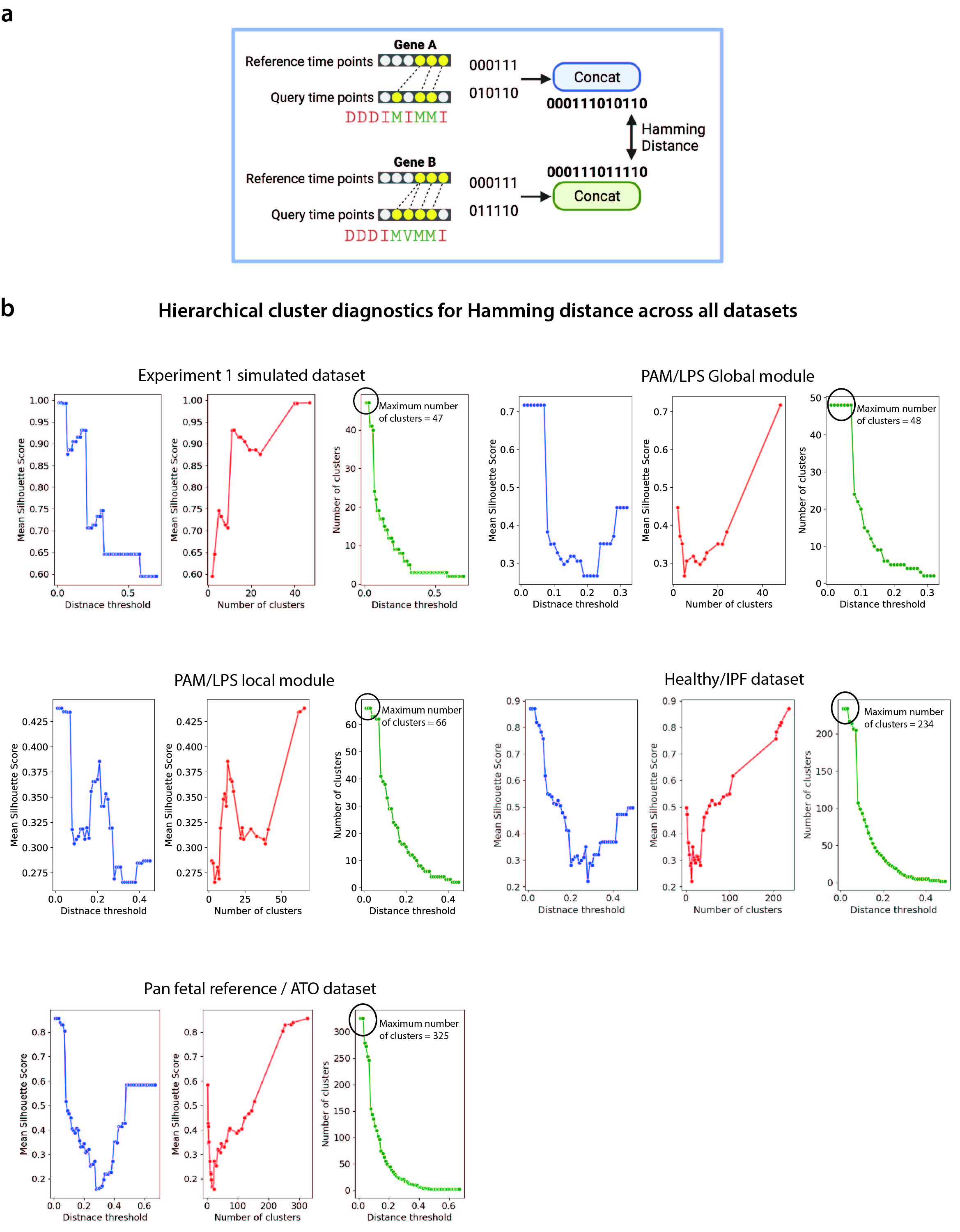

Supplement: Supplementary file 4 — G2G alignment data and Supplementary Fig. files. [file 41592_2024_2378_MOESM4_ESM.zip › SupplementaryData/SupplementaryFigures/SupplementaryFig6.jpg]

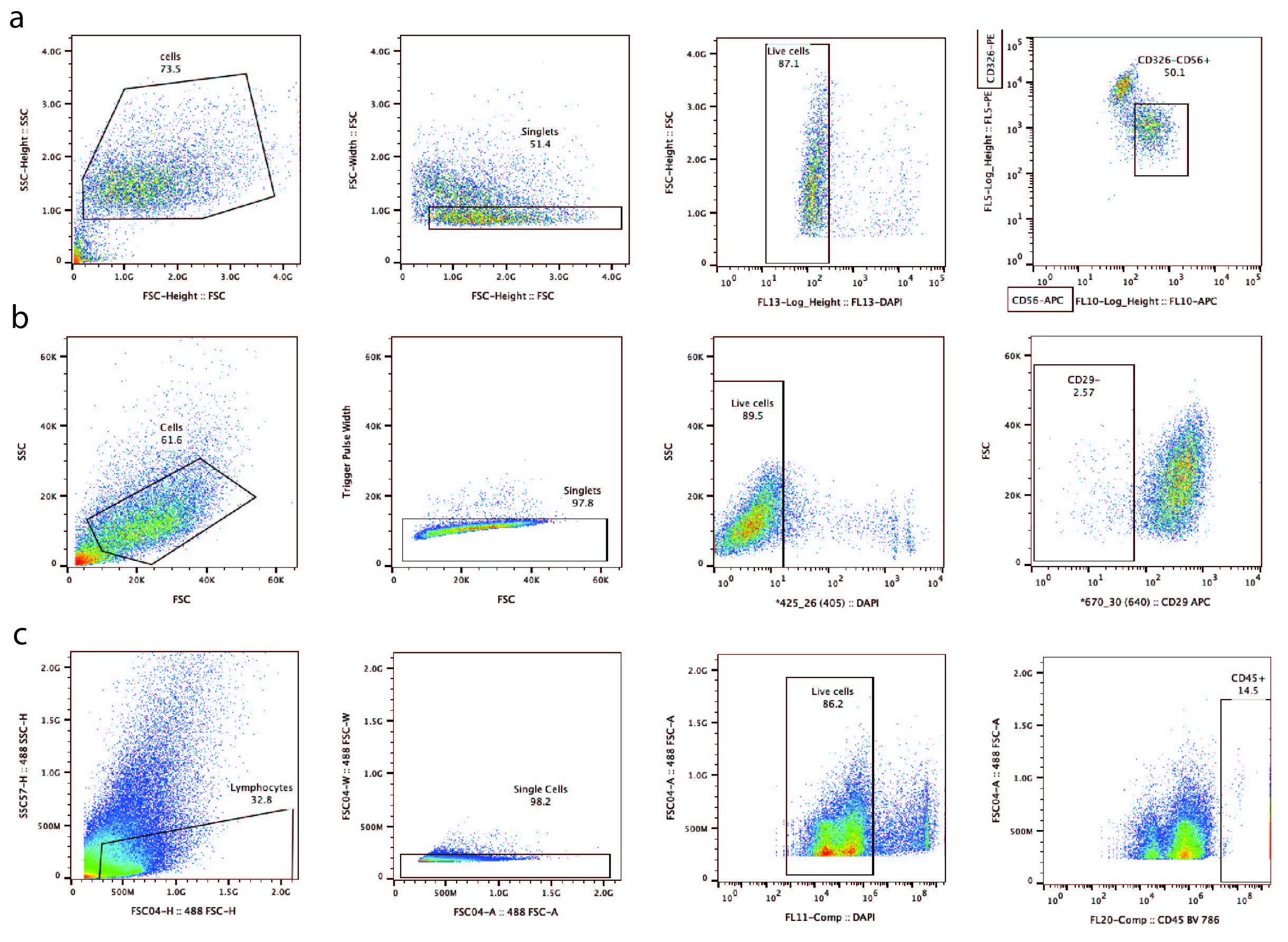

Supplement: Supplementary file 4 — G2G alignment data and Supplementary Fig. files. [file 41592_2024_2378_MOESM4_ESM.zip › SupplementaryData/SupplementaryFigures/SupplementaryFig7.jpg]

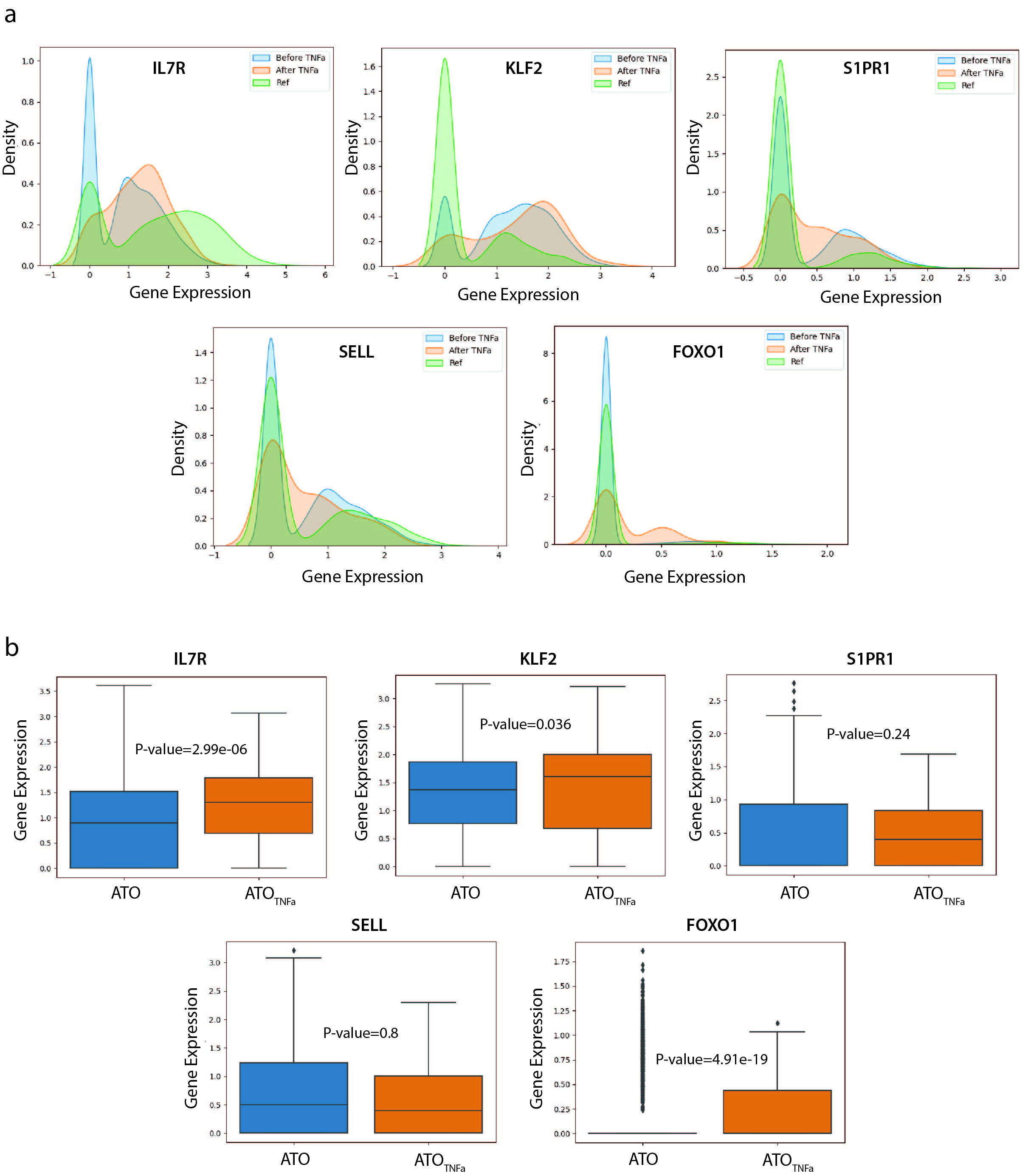

Supplement: Supplementary file 4 — G2G alignment data and Supplementary Fig. files. [file 41592_2024_2378_MOESM4_ESM.zip › SupplementaryData/SupplementaryFigures/SupplementaryFig5.jpg]

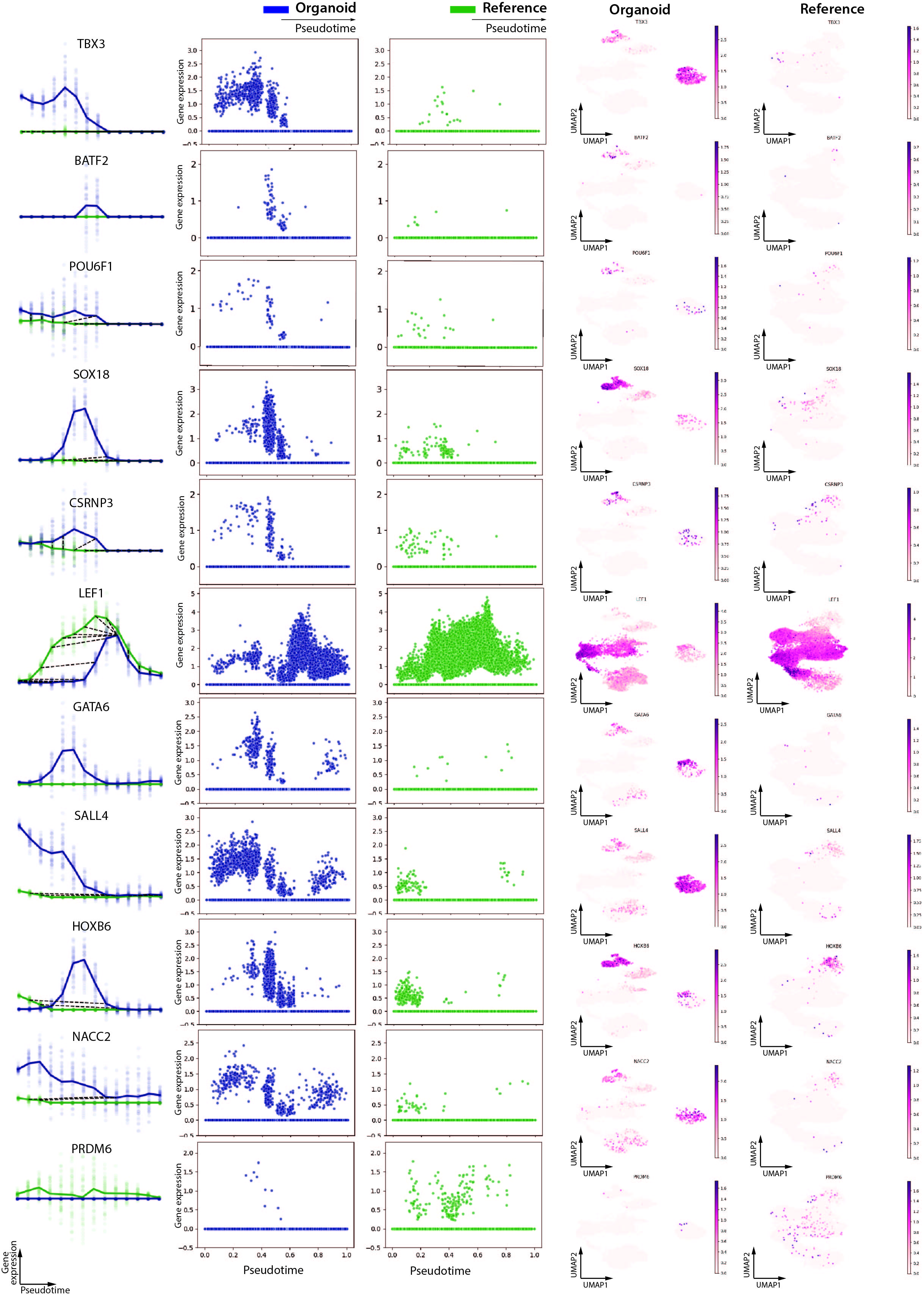

Supplement: Supplementary file 4 — G2G alignment data and Supplementary Fig. files. [file 41592_2024_2378_MOESM4_ESM.zip › SupplementaryData/SupplementaryFigures/SupplementaryFig4.jpg]
